# Nano-motion Dynamics are Determined by Surface-Tethered Selectin Mechanokinetics and Bond Formation

**DOI:** 10.1371/journal.pcbi.1000612

**Published:** 2009-12-18

**Authors:** Brian J. Schmidt, Jason A. Papin, Michael B. Lawrence

**Affiliations:** Department of Biomedical Engineering, University of Virginia, Charlottesville, Virginia, United States of America; Medical College of Wisconsin, United States of America

## Abstract

The interaction of proteins at cellular interfaces is critical for many biological processes, from intercellular signaling to cell adhesion. For example, the selectin family of adhesion receptors plays a critical role in trafficking during inflammation and immunosurveillance. Quantitative measurements of binding rates between surface-constrained proteins elicit insight into how molecular structural details and post-translational modifications contribute to function. However, nano-scale transport effects can obfuscate measurements in experimental assays. We constructed a biophysical simulation of the motion of a rigid microsphere coated with biomolecular adhesion receptors in shearing flow undergoing thermal motion. The simulation enabled in silico investigation of the effects of kinetic force dependence, molecular deformation, grouping adhesion receptors into clusters, surface-constrained bond formation, and nano-scale vertical transport on outputs that directly map to observable motions. Simulations recreated the jerky, discrete stop-and-go motions observed in P-selectin/PSGL-1 microbead assays with physiologic ligand densities. Motion statistics tied detailed simulated motion data to experimentally reported quantities. New deductions about biomolecular function for P-selectin/PSGL-1 interactions were made. Distributing adhesive forces among P-selectin/PSGL-1 molecules closely grouped in clusters was necessary to achieve bond lifetimes observed in microbead assays. Initial, capturing bond formation effectively occurred across the entire molecular contour length. However, subsequent rebinding events were enhanced by the reduced separation distance following the initial capture. The result demonstrates that vertical transport can contribute to an enhancement in the apparent bond formation rate. A detailed analysis of in silico motions prompted the proposition of wobble autocorrelation as an indicator of two-dimensional function. Insight into two-dimensional bond formation gained from flow cell assays might therefore be important to understand processes involving extended cellular interactions, such as immunological synapse formation. A biologically informative in silico system was created with minimal, high-confidence inputs. Incorporating random effects in surface separation through thermal motion enabled new deductions of the effects of surface-constrained biomolecular function. Important molecular information is embedded in the patterns and statistics of motion.

## Author Summary

The binding of a receptor on one cell to a ligand on another is a process of broad biological interest, important to cell adhesion and signaling. Interactions between cell surfaces can be called “two-dimensional” because the reactive groups on interacting molecular pairs are constrained to move 100 nm or less in the direction perpendicular to the surfaces. The molecular reactive groups are attached to their respective cellular surfaces through a molecular tether embedded in the cell membrane. There are many parameters that might affect the observed binding kinetics, such as the distance between the cell surfaces, the length of the molecular tether, and the freedom of the reactive groups to move about on their molecular tether. A well-studied case of two-dimensional interactions is that through which circulating leukocytes capture to the endothelium and exit the blood into the tissues. Leukocyte capture presents an additional complexity: bonds that restrain leukocytes against the shearing force exerted by the blood must be capable of withstanding the force trying to pull the receptor and ligand apart at their noncovalent interface. New models have been proposed to explain the behavior of individual receptors and ligands, raising the question: which molecular behaviors have an effect on function?

## Introduction

Cell-cell interactions are critical in a variety of biological processes such as morphogenesis, immune responses, and homing. The interaction of surface-tethered biomolecules between cells is essentially two-dimensional because of the limited ability of the molecules to move in the dimension perpendicular to the cell surfaces. Receptors and ligands must therefore find each other by lateral motion on their respective surfaces [1]. A reactive head group attached to a macromolecular stalk extending from the surface of a cell results in a configuration with more factors governing function and more effects on cellular behavior than with the three-dimensional, freely-diffusive case. For example, in vascular homing the force response of the surface-tethered molecules is critical [2]. In cytotoxic T-cell mediated apoptosis, the two-dimensional, sorted arrangement of interacting partners might be important to developing a long-lived, death-mediating signaling complex [3].

Striking behaviors result from the complexity of surface-tethered molecules. The existence of catch-slip bonds has recently been demonstrated [4]–[6]. Catch-slip bonds exhibit the unexpected property that, as the force transmitted through the binding pocket increases, bond lifetimes increase prior to reaching a peak and then decrease. It has also been demonstrated that tether-constrained molecules more efficiently form bonds in some presentation contexts [7]. A two-dimensional molecular interaction system that has been studied is the binding of T-cells to antigen-presenting cells. There are many proteins involved in the intercellular interaction, and the binding of CD2 with LFA3 has been studied. As the membranes between the two cells remain in contact, they smooth against each other and form a space with a small separation [8]. Constraining the most likely position of the reactive sites to overlap well within the volume between the cells, or an even smaller space within the volume between the cells [9], was found to result in up to a 40-fold enhancement in the effective reaction rate [8].

The molecular characteristics, such as length, flexibility of the molecular tether, and the binding pocket chemistry, that facilitate bond formation for two-dimensional interactions when the contact between surfaces is less than one second may be very different from the molecular characteristics that facilitate adhesion when the contact lasts minutes to hours. One example is cells traveling through the blood that capture to a blood vessel surface as a first step in homing to tissue. Vascular homing processes occur as white blood cells are recruited to sites of inflammation, lymphocytes travel to the lymph nodes, cancer cells metastasize to spread to new tissues, and stem cells home to sites of injury to repair tissues [10]–[12]. A cell traveling in excess of hundreds of cell diameters a second may briefly bump into the wall, leaving no opportunity for the proteins in the membrane of the flowing cell and in the membrane of the immobilized cell on the vessel wall to adapt for an optimal, sorted presentation of molecules. Although the average density of CD2 on T-cells is around 200 molecules per square micrometer [13], within a factor of two of the average density of ligands mediating capture and dynamic adhesion on neutrophils [14], the adhesive contacts involving hundreds of molecules per square micrometer have been observed to require thirty minutes to fully form in vitro with assays for CD2 and LFA3 [7]. T-cell interactions with antigen presenting cells have been observed to go through several phases in vivo, involving contacts lasting a few minutes and contacts lasting hours [15], suggesting molecular sorting in the contact region plays a role in vivo. On the other hand, dynamic adhesion ligands are thought to be localized cellular membrane ruffles called microvilli [16]. The contact widths and times for these ridges during cell capture are much shorter and smaller than for CD2 and LFA3, as small as 100 nm and as short as 1 ms [16],[17], respectively. Also, once they form, the reacting pairs must survive higher forces exerted on the cell. Consequently, there may be a specialized set of structural, dynamic, and kinetic features of the molecules responsible for cell capture that facilitate rapid molecular tether formation and lifetime.

Selectins mediate dynamic interactions between cellular surfaces. Selectins have received considerable attention because of their importance in inflammatory and immune trafficking as well as their role in diseases such as atherosclerosis and cancer metastasis [10],[18]. Many assays have been employed to make measurements of selectin molecular interactions: laser traps, atomic force microscopy, biomembrane force probes, and flow cells [5], [19]–[23].Arguably, a significant advantage of flow cells is that they give a report of molecular binding that is quite functionally relevant. Flow cell assays capture the characteristics of a hydrodynamic environment more directly than single-molecule assays. They balance the experimental complexity of an in vivo vascular model and the ability to make deductions about biomolecular interactions at the most basic level. Observations of complicated cellular behavior in a flow cell, such as hydrodynamic shear thresholding, have helped to inspire the application of force spectroscopy techniques that have established the existence of catch-slip bonds [24],[25].

It is not clear which known qualitative molecular characteristics are important to their functional ability to capture a cell or particle and initiate bonds that can withstand detaching forces. We therefore adapted an adhesive dynamics modeling strategy that can test functionally relevant P-selectin/PSGL-1 molecular behaviors.

Novel aspects of the simulation and analysis methodology were:

- The simplest biophysical experimental system capable of reporting biomolecularly-dependent behaviors was simulated. This is the microbead assay. Simplicity minimized the number of simplifying assumptions that were made and minimized sources of error and uncertainty.
- Thermal motion of the sphere was included in the model.
- The adhesion receptors were modeled as extending from discrete points on the surface of the sphere. The modularity made it simple to change the form of the rate expression for bond formation to test the effects of the dependence of bond formation on surface separation. The concept is intricately linked to molecular confinement, whereby the rate of reaction between a receptor and ligand is enhanced by physically constraining their reactive end groups to more efficiently make contact.
- A detailed analysis of the simulated sphere's motions was made. Analyzed motion characteristics included pause times, distances between pause events (skip distances), and the autocorrelation of velocity perpendicular to the flow direction (wobble autocorrelation). A detailed qualitative analysis of the motions was also performed.

The simulation results and analysis methodology resulted in several new findings:

- Clustering adhesion molecules into functional groups that equally distribute the load is critical for function. Nano-scale molecular clustering reconciles results from different assays. Clustering makes it possible to capture the sphere and create “stop and go” motion at physiologic ligand densities despite large predicted forces on the cluster.
- Initial, capturing bond formation effectively occurred across the entire molecular contour length, although rebinding events were enhanced by vertical transport to the wall through initial capture.
- The wobble velocity autocorrelation is proposed as new metric to verify how molecules behave at the interface between surfaces and validated in silico. The wobble velocity autocorrelation may help uncover molecular behaviors not previously investigated but perhaps important for the function of additional classes of molecules.

There were also two findings where qualitative changes in the expression governing molecular behavior did not make a difference to the simulated sphere's motion. The effects of molecular confinement on bond formation were not functionally important to dynamic adhesion. The result demonstrates a criterion where successful static and dynamic interaction systems differ. Also, P-selectin/PSGL-1 catch-slip bonds performed nearly as well as slip-only bonds in mediating capture interactions, and were equally effective at mediating pauses. The result reinforces the hypothesis that a purpose for catch bonds might lie in distinguishing soluble ligands from immobilized ones rather than regulating the dynamics of adhesion, at least for P-selectin/PSGL-1.

The implications of the present study are extensive. With an increased understanding of the molecular features that enhance bond formation between surfaces, it will be possible to engineer molecular systems with an optimal physiologic impact. Optimizable systems include enhanced targeted drug delivery and molecular imaging agents and dendritic cell therapies with the potential for enhanced T-cell activation.

## Methods

**Figure 1:**
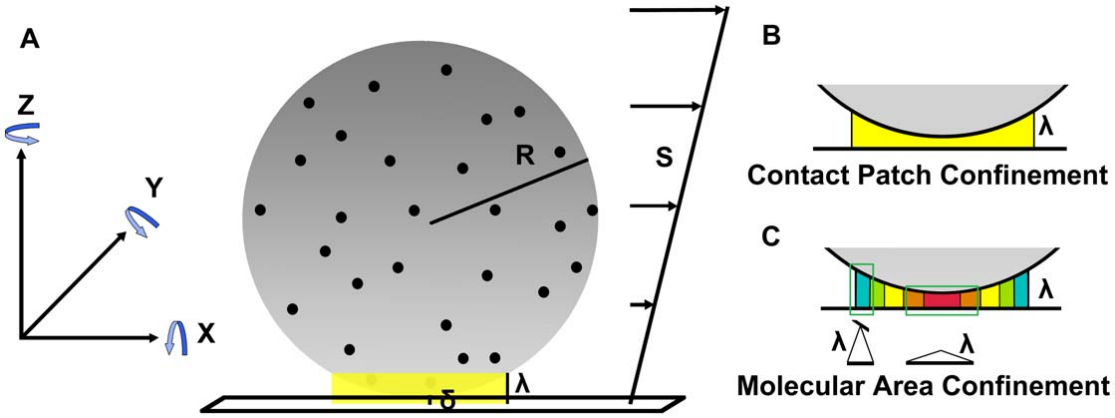
Simulation geometry. (A) A Cartesian coordinate system was employed. Flow was applied in the X-direction with a linear shear rate, S. A three dimensional sphere with a fixed radius, R, was coated with receptors, and each anchor point of the base of the receptor's tether region to the surface of the sphere is shown as a black dot. Only the receptors within an unstressed receptor/ligand contour length of the surface, λ, were allowed to form bonds. This region has been highlighted in yellow. The gap between the base of the sphere and the surface, δ, was allowed to vary. The diffusion of the sphere was included in the simulation. The diffusion had six components with the inclusion of rotation in the sphere's motion. Motion perpendicular to the flow direction, along the Y-axis, is referred to as “wobble” in the text. (B,C) Two different models of reactivity for molecules in the contact volume were included in the simulations. For contact patch confinement, described mathematically by (1), all of the receptors on the sphere within an unstressed receptor/ligand contour length of the surface were assumed to react with a constant rate. For molecular area confinement, described mathematically by (2), receptors immobilized on portions of the sphere closest to the surface were allowed to form bonds with an increased rate proportional to the area of the projection of the molecular contour length onto the XY plane. The colors in (B,C) depict the relative reaction rate of receptors, and warmer colors indicate an increased reaction rate.

**Figure 2:**
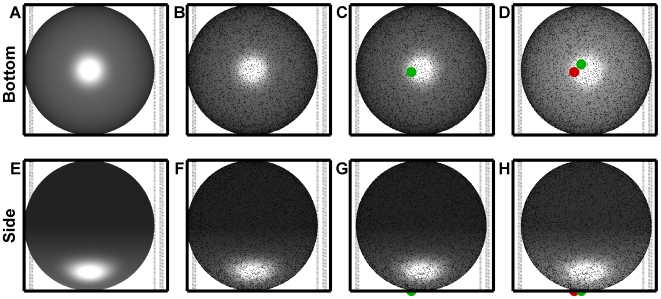
Iterative calculations in the simulation. (A,E) An initial gap size, generally small compared to the sphere's radius, was randomly selected from the governing equilibrium distribution. (B,F) Receptors were randomly distributed over the surface. Fluid flow was started. Forces and torques were calculated, a diffusive component was added, the new sphere position and rotation was calculated, and receptors were tested for bond formation. (C,G) The sphere was translated and rotated into the new position. One bond formed in the calculation from the previous step. New force calculations on the bonds and sphere were performed. The bond's green color indicates the sphere had not yet moved enough to begin mechanically stressing the bond. The new sphere position and rotation was calculated based on the forces and toques plus the diffusive component, then free receptors were tested for formation, and the existing bond was tested for breakage with the current position. (D,H) The sphere was translated and rotated into the new position. The brighter sphere coloring indicates the sphere moved closer to the ligand-coated surface. Vertical motion was significant compared to the length of a bond. The bond from the previous step was still present, and a new bond formed. If the new bond were to survive until it became stressed, it would exert a force perpendicular to the flow direction that will cause the sphere to wobble because it is off the sphere's center line. Force calculations on the bonds and sphere were performed. The red color indicates the trailing bond was stressed and exerted forces and torques on the sphere. Next, the diffusive component would be added to the forces and torques on the sphere, the new sphere position and rotation would be calculated, then free receptors would be tested for formation and the existing bonds would be tested for breakage. The position would be updated and the calculation iterated.

For each simulation, discrete attachment points for the base of individual adhesion receptor molecules were randomly distributed over the surface of a microsphere. To accurately capture scenarios where the adhesion receptor was immobilized to the surface, as with experimental microbeads or proteins anchored to the cytoskeleton, on-sphere diffusion of the anchorage points was assumed to be zero. A stochastic methodology was employed to include three-dimensional lateral, vertical, and rotational diffusion of the sphere and is described in greater detail in Protocol S1. Three-dimensional diffusive motion of the molecular binding pockets within the contact volume was treated during consideration of the on-rate expression. The simulation geometry is illustrated in Figure 1, and steps in the calculation are detailed in Figure 2.

Two models were compared to account for different mobilities of the molecular binding pockets on their respective tethers. In the first, it was assumed each receptor within a bond length of the surface could find a ligand with an equal rate:
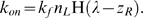
(1)


The symbols and values employed are defined in Table 1. The Heaviside function, denoted by H( ), limited nonzero bond formation rates to receptors in the contact patch. The reaction rate described by (1) could be described as contact patch confinement because only receptors in the contact patch could react, and finer details of the reaction configuration were assumed to be unimportant for receptor function. The concept is illustrated in Figure 1B.

**Table 1:** Symbols used in the text and parameter values used in the simulation.

Symbol	Description	Value	Units	Reference
γ	Slip bond compliance	0.37 or 1	Å	[23],[48]
γ_20_	High impedance catch-slip compliance	2.4 or 0.68	Å	[22]; regression in [32] for [5]
γ_21_-γ_12_	Difference in catch-slip bond compliances for conformation transitions	8.7 or 12	Å	[22]; regression in [32] for [5]
δ	Gap between the sphere and the wall	Variable	∼10–200 nm	Protocol S1
δ_min_	Surface roughness	10	nm	Near ideal surfaces
Δt	Time step size	Variable	∼µs	Protocol S1
λ	Maximum unstressed bond length	92	nm	[36],[37]
ρ_s_	Sphere density	1.05	g/mL	Manufacturer web site
ρ_w_	Water density	1.00	g/mL	
σ	Bond spring constant	100 or 5.3	pN/nm	Rope bonds [34],[35]; freely-jointed chain bonds [21]
Φ_o_	Catch-slip bond probability ratio of low to high impedance state when unstressed	90 or 21.7	Ratio	[22]; regression in [32] for [5]
g	Gravitational constant	9.8	m/s^2^	
k_10_^o^	Catch-slip bond dissociation rate for the low impedance pathway	10 or 5.39	s^−1^	[22]; regression in [32] for [5]
k_20_^o^	Catch-slip bond unstressed dissociation rate for the high impedance pathway	0.37 or 1.66	s^−1^	[22], or regression in [32] for [5]
k_B_	Boltzmann constant	1.38×10^−23^	J/K	
k_f_	Bond formation rate between two surfaces per unit area per unit site density receptor and ligand	4.8×10^−4^	µm^2^/s	Deduced from [60]
k_f_^T^	Bond formation rate between two surfaces in very close proximity	∼1.7×k_f_	µm^2^/s	Protocol S1
k_on_	Rate at which a receptor finds a ligand on the surface and forms a bond	Variable	s^−1^	(1,2)
k_r_	Bond dissociation rate	Variable	s^−1^	(3,4)
k_r_^o^	Unstressed slip bond dissociation rate	1.54 or 0.53	s^−1^	[23] or [48]
M	Mobility matrix	Varies	Varies	Protocol S1
n_L_^o^	Total density of individual ligands or clusters	100 or 90	sites/µm^2^	90 to match [23]
n_L_	Density of unbound ligands in the contact patch	Variable	sites/µm^2^	
n_R_°	Density of individual receptors or receptor clusters on the sphere	50 or 95	sites/µm^2^	95 to match [23]
N_RL_	Number of receptor-ligand complexes	Variable	number	
p	Persistence length of freely-jointed chain	3.5	Å	[21]
R	Sphere radius	3, 5, or 4.9	µm	4.9 to match [23]
S	Wall shear rate	50 or 100	s^−1^	50 to match [23]
T	Temperature	295	K	
V_S_	Sampled instantaneous velocity	Variable	µm/s	
V_S,X_	Sampled flow-direction instantaneous velocity	Variable	µm/s	
V_S,Y_	Sampled perpendicular-direction instantaneous velocity	Variable	µm/s	
z_R_	Z-coordinate of the anchorage point of a receptor	Variable	nm	

In the second approach, receptors with anchorage points on the sphere closer to the surface were assumed to sample a wider area of the surface, enhancing their probability of encountering an immobilized binding partner:
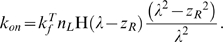
(2)


We refer to the reaction model described by (2) as molecular area confinement. The geometry of the search by the receptor's binding pocket for a ligand immobilized within a suitable distance influences the reaction. Therefore, (2) is conceptually similar to the geometric interpretation of the enhanced apparent association rate of laterally diffusive cellular CD2 with LFA3 on two surfaces with an extended contact time [7]. According to (2), reactions proceed more quickly when the receptor is held closer to the surface and sweeps out a broader area in the search for ligands. The concept is illustrated in Figure 1C. The rate, k_f_^T^, was normalized based on k_f_ so both (1) and (2) would yield the same average reaction rate across the contact patch for a sphere touching the wall to within surface roughness limitations.

A number of models of receptor-ligand dissociation kinetics under an applied load have been developed to describe the dissociation of selectins from their ligands. One of the first models, based on observations of non-covalent solid materials failure [26], proposed that the application of force increases the dissociation rate in an exponential manner [1]. Bonds exhibiting this type of force response kinetics are referred to as “Bell slip bonds” to distinguish the model from alternate proposals for the forced dissociation relation [27]–[29]. Increasing force causes the slip bond to dissociate more quickly. Newer measurement techniques demonstrated force decreases selectin bond dissociation rates until a peak mean lifetime is reached, and higher levels of force increase the bond dissociation rate. Bonds exhibiting this type force response kinetics are referred to as “catch-slip” bonds because of the biphasic force response. Several theoretical and mathematical models have been developed to describe catch-slip bonds [22],[25],[30],[31]. The five-parameter model of rapid internal state equilibration [22] is an appealing model that has a sound theoretical basis, captures the salient quantitative features of selectin dissociation kinetics [32], and converges on a high-impedance dissociation pathway at high forces that closely resembles the Bell slip bond model of dissociation.

The dissociation rate for existing receptor-ligand complexes was first computed using the Bell slip bond model for dissociation under an applied load [1],[26]:
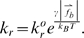
(3)


After initial validation and investigation with slip bonds, the effect of catch-slip bonds on motion was investigated utilizing the five-parameter model of rapid internal state equilibration [22]:
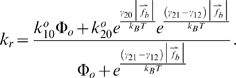
(4)


It was assumed a bond exerted no force when in compression but behaved as a very stiff spring when stretched past its contour length. A constant dissociation rate for a bond in compression has been assumed in previous simulations of selectin-mediated rolling [33]. We refer to the model combining this assumption with a stiff spring for extension [34],[35] as a rope model. The expression employed was:
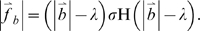
(5)


Note that the rope model spring constant was very high, so bonds did not extend much past their equilibrium length. Alternatively, a freely-jointed chain model was used with the condition that a chain in compression exerted no force:
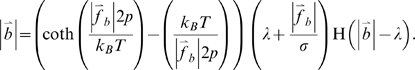
(6)


Although the time steps were much smaller, data from the model was sampled to file at 1,000 fps, in analogy with experiment. This represents an upper sampling limit for many experimental systems used to acquire data optically. Numerical parameter values used in the simulation are given in Table 1. The force deformation models are described in greater detail in Figure S1.

## Results

### Validation of motion relations with non-interacting microspheres

**Figure 3:**
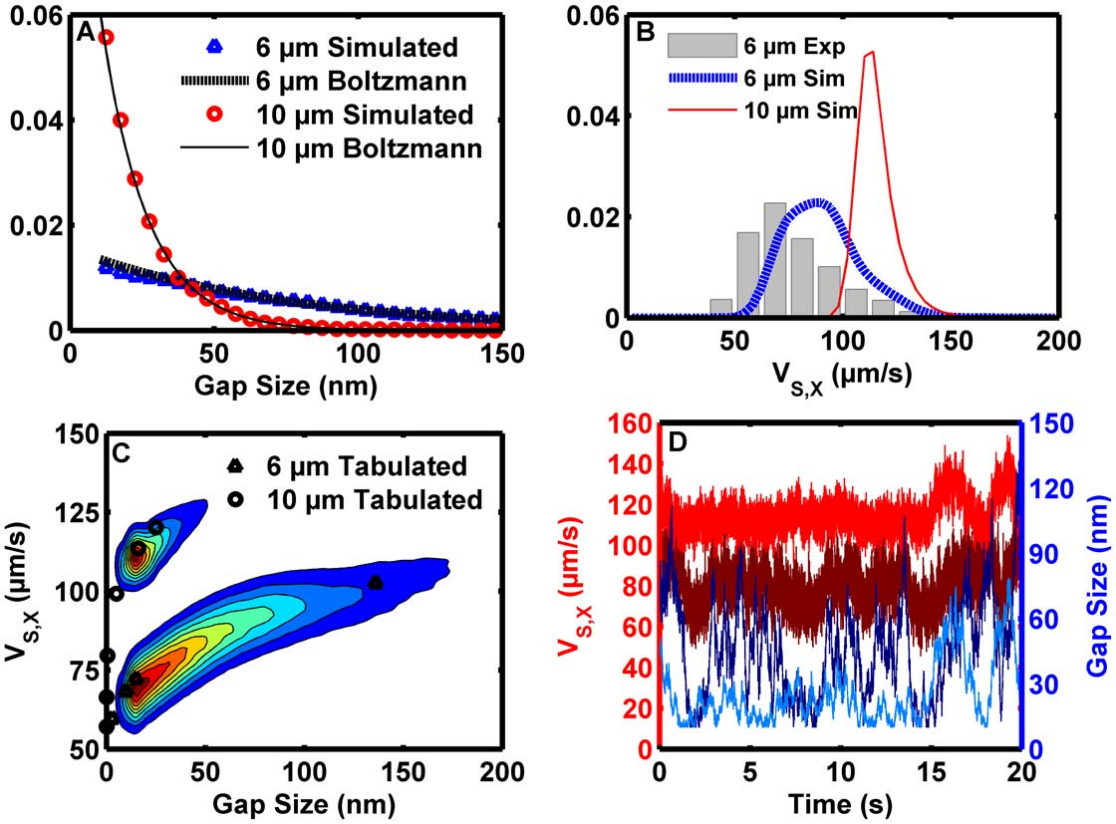
Investigation of non-reactive microsphere motion with a 50 s^−1^ wall shear rate. (A) The probability distribution functions for gap size for a simulated 6 µm-diameter sphere (blue triangles), the theoretical Boltzmann distribution for a 6 µm-diameter sphere (heavy dashed line), a simulated 10 µm-diameter sphere (red circles), and the theoretical Boltzmann distribution for a 10 µm-diameter sphere (narrow solid line) are shown. The simulation results agree well with the equilibrium theory and demonstrate vertical diffusion occurs over a biochemically relevant length. (B) Sampled instantaneous, flow-direction velocity probability distribution functions for a 6 µm-diameter sphere (heavy blue dashed line) and a 10 µm-diameter sphere (narrow red line) are shown and compared to the experimental results with microbeads possessing a nominal diameter of 6 µm [39] (grey bars). The results demonstrate the experimentally observed skewing of the instantaneous velocity distribution from normal and predict a tighter velocity distribution for larger particles. (C) Contour plots of the probability distribution functions of the sampled instantaneous, flow-direction velocity for a simulated 6 µm sphere (bottom) and a 10 µm simulated sphere (top) at 50 s^−1^ are shown. Each was re-normalized to the respective maximum, so the results for the 6 µm-diameter sphere cover a larger area. Tabulated deterministic solutions published by Goldman et al. [40] are shown as triangles (6 µm-diameter sphere) and circles (10 µm-diameter sphere). Although agreement was good, the simulation slightly underpredicted the superposition result. (D) Time-domain flow-directed instantaneous velocity for a 6 µm-diameter sphere (maroon line), flow-directed instantaneous velocity for a 10 µm-diameter sphere (red line), gap size for a 6 µm-diameter sphere (dark blue line), and gap size for a 10 µm-diameter sphere (light blue line) are shown. The low-frequency fluctuations in the instantaneous velocity reflected fluctuations in the gap.

Sphere motion in the absence of binding interactions established a baseline for both validation against experimental results and comparison to reactive sphere motion. Vertical stepping accuracy was first investigated by recording the gap and velocity distribution of vertically diffusing microspheres over a long time interval in the presence of gravity and fluid flow. Figure 3 compares the results for motion between a 6 µm-diameter and a 10 µm-diameter sphere in the presence of a 50 s^−1^ wall shear rate for 1,000 s of simulated time. The results for vertical motion are shown in Figure 3A. As in all simulations presented here, a minimum separation distance of 10 nm was enforced with a reflective boundary condition to simulate a minimal wall roughness that would be experimentally achievable and avoid singularities in the fluid dynamic equations. The data for the simulated microspheres agreed very well with the theoretical Boltzmann distribution for particles diffusing in a gravitational force field, validating the vertical stepping method.

Thermal fluctuations in the vertical coordinate were more significant for smaller particles than for larger ones. The length scales for vertical motion in Figure 3A are biochemically relevant because biomolecular interactions occur on the nano-scale. Of special interest is the PSGL-1/P-selectin bond, which has been estimated to extend up to roughly 90 nm when unstressed [36]–[38]. The simulation results predict the 10 µm-diameter and 6 µm-diameter spheres will remain with 90 nm of the surface 99% and 67% of the time, respectively, in agreement with the Boltzmann distribution. Only after 1,000 s of simulation time did the probability distribution function converge well upon the equilibrium distribution. The long time required to achieve statistical equilibrium suggested the importance of transport history for interacting spheres. The long equilibration time has implications for experimental flow cell studies employing microbeads. First, all of the beads that appear to be near the wall are not equivalent. Some beads spend a larger portion of their time near the surface than others while traversing a field of view. Some beads are sufficiently far so they are incapable of binding even if the population is given sufficient time for sedimentation. Secondly, a flow cell must be very long, 10 cm, for the beads in a field of view to follow the equilibrium distribution if it takes 1,000 s for a 10 µm-diameter bead to be able to fully sample the gap possibilities once the bead sediments to near the surface.

The probability distributions for the sampled flow-direction velocity, V_S,X_, for the non-interacting spheres flowing at 50 s^−1^ are shown in Figure 3B. Variation in the convective component of the motion was expected due to variations in the vertical coordinate of the sphere. It is stressed that these are sampled velocities: there was a convective as well as an effective diffusive component to motion in the flow direction. As the gap between the bottom of the sphere and the wall increased, the sphere's velocity increased due to the linear shear gradient. The apparent flow-plane diffusive component of the sampled velocity may be positive or negative. The standard deviation of the diffusive component of the sampled velocity will decrease as the sampling rate is decreased (refer to Protocol S1). As might be expected, the mode of the sampled velocity data for the 6 µm-diameter sphere was lower than the mode for the 10 µm-diameter sphere due to the smaller size. The sampled velocity for non-interacting, 6 µm-diameter spheres flowing at 50 s^−1^ was skewed from normal, very similar to the pooled experimental instantaneous velocity results as previously reported [39]. The mode of the simulation results, 90 µm/s, was higher than the experimental mode previously reported [39], 70 µm/s. The discrepancy was still within a range that can be accounted for by experimental differences in the microbead size, possible variations in the observed populations, and differences in the surface roughness and coatings. The sampled velocity results for the 10 µm-diameter sphere were less skewed because there was a smaller variation in the distribution of gap size for the larger sphere.

A statistical plot of V_S,X_ against the gap size is presented in Figure 3C for the 6 µm-diameter and 10 µm-diameter spheres. During each time step, the forces, torques, and damping factors acting on the sphere were computed by interpolating the individual fluid dynamic solutions and then applied using superposition. The deterministic superposition solutions previously tabulated [40] are shown on the same plot. There was a slight disagreement between the statistical mode for the simulation and the tabulation. Observing vertical slices through the “Gap Size” axis, it is apparent that the mode of V_S,X_ as a function of gap size is less than the tabulated value reported by Goldman et al. [40]. There are two possible reasons for the discrepancy. The first may be a cumulative effect of residual errors in the interpolation method used to obtain the individual hydrodynamic damping factors. The other is that the statistical weighting of motions truly results in a mode that is lower than the deterministic solution. Such differences have been theorized to occur with important consequences in biomolecular reaction systems [41]. Despite the minor discrepancy, the results agreed sufficiently for present purposes. Figure 3D presents sample time domain V_S,X_ data for the both sphere sizes. The vertical excursions of the 10 µm-diameter sphere away from the wall were infrequent and of small magnitude. The high-frequency fluctuation in V_S,X_ was largely due to the high sampling rate: lateral diffusion was well distributed across frequencies. The low-frequency component of the velocity fluctuations agreed well with variations in the gap size.

### Validation of motion with binding microspheres: rope vs. freely-jointed chain

**Figure 4:**
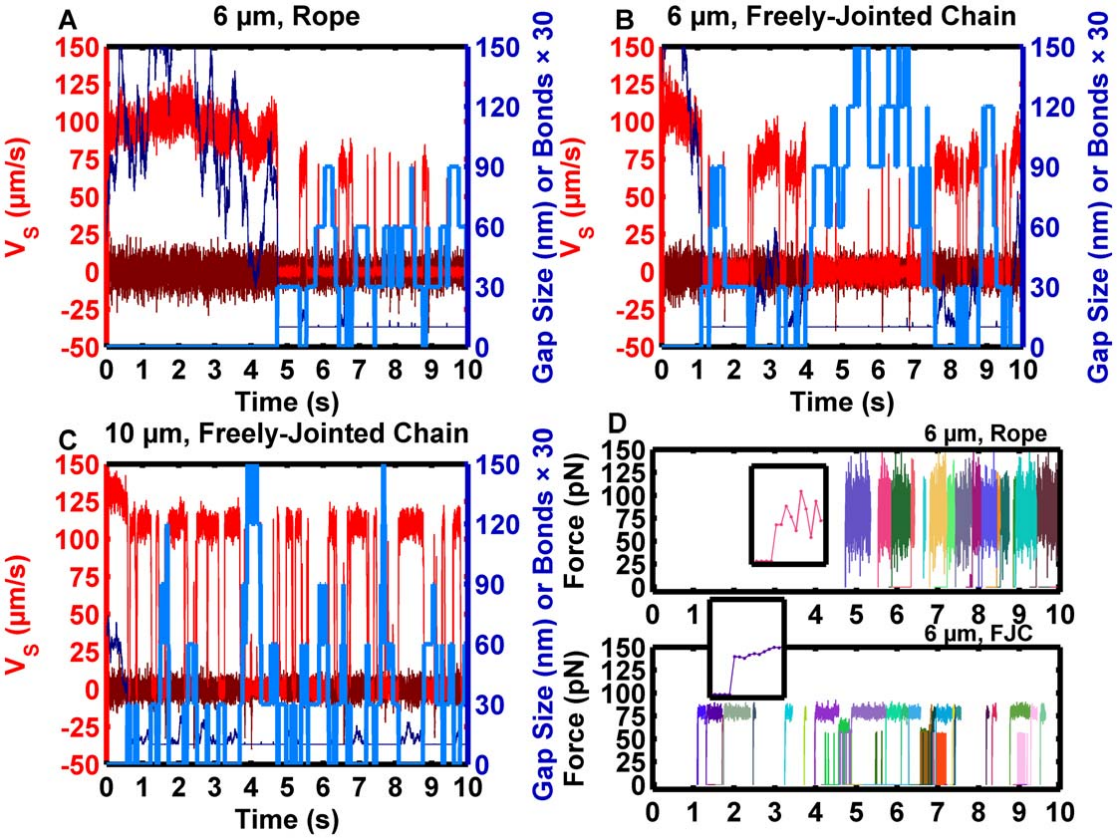
Reactive microsphere motion illustrates the discrete nature of bond formation events and force loading. Reactive spheres were simulated with S = 50 s^−1^, n_L_° = 100 sites/µm^2^, n_R_° = 50 sites/µm^2^, association kinetics governed by (1), and dissociation kinetics governed by (3). (A) Results for a 6 µm-diameter sphere with a rope model of bond deformation, (5). Sampled flow-direction velocity (V_S,X_, red), perpendicular velocity (V_S,Y_, maroon), gap size (dark blue) and number of bonds (light blue) are shown. (B) Results for a 6 µm-diameter sphere with a freely-jointed chain model of force deformation, (6). An increase in the magnitude of the fluctuations of V_S,X_ was observed with the decrease in stiffness relative to (A). (C) Results for a 10 µm-diameter sphere with a freely-jointed chain model of force deformation, (6). The motion was higher frequency in nature with shorter pauses and more frequent pause events. (D) A comparison of simulated bond loadings for the rope tether results in (A), top, and the freely-jointed chain in (B), bottom. Each sampled point on the inset is spaced by 1 ms. In both cases, the sphere's pause tended to be supported by a singly-loaded bond. The decreased stiffness of the freely-jointed chain tether resulted in a smaller standard deviation of the supported force. A lower maximum initial force loading was predicted for the freely-jointed chain model than for the rope model (insets).

The effects of microsphere size and the molecular force distension model were compared to gain a quantitative understanding of how contact area and molecular distension affect motion. Figures 4A–C present simulation results for spheres bearing 50 sites/µm^2^ of receptor interacting with 100 sites/µm^2^ of ligand at a wall shear rate of 50 s^−1^. The data for Figure 4 was sampled at 1,000 fps. Figure 4A presents simulation results for a 6 µm-diameter sphere that forms rope-like bonds, Figure 4B presents data for a 6 µm-diameter sphere that forms freely-jointed chain bonds, and Figure 4C presents data for a 10 µm-diameter sphere that forms freely-jointed chain bonds. As an additional tool to validate and interpret the physics, three-dimensional videos were constructed from the simulation results (sampling reduced to 250 fps, Videos S1, S2, S3). One second of data was selected from each of these scenarios when constructing the videos. The videos illustrate the microsphere behaved in a physically realistic way. Bond formation events introduced realistic forces and torques that caused the sphere's rotational orientation and centroid to converge on a stable mechanical equilibrium point. The sphere was still free to undergo stochastic fluctuations in translation and rotational orientation when settled in the bound state. Roughness held the sphere 10 nm from the absolute, mathematical surface, but did not prevent the sphere's oscillations. In practice, the motion of a bead being pushed into the surface by a biomolecular lever arm depends on the details of the experimental surface construction and blocking strategy. The results must be construed as a case representing an ideal experimental methodology.

The results recreated the discrete “stop and go” behavior observed with microbeads and demonstrated the effect of differences in biomolecular tether properties. Discrete stops were apparent in Figures 4A–C as drops to a zero-mean, fluctuating V_S,X_. The results closely capture the discrete pause behavior reported for selectin-coated microbeads [39]. The results also illustrated differences in molecular tether stiffness can influence the sphere's motion during a binding event. It is important to point out the interpretation of experimental velocity fluctuations must also include a consideration of noise in the acquisition system [42]. Fluctuations in V_S,X_ in for the sphere bound by the freely-joined chain in Figure 4B were larger than the sphere bound by the rope in Figure 4A, although fluctuations in V_S,Y_ agreed more closely. Although there were significant fluctuations in the gap between the bottom of the sphere and surface prior to the first bond formation event, the sphere maintained contact following the first capture event.

Multiple bonds were often present, but most frequently only one bond supported the sphere against the hydrodynamic load. The bond loading forces from the trials in Figures 4A,B are presented in Figure 4D. The top panel illustrates the result with the rope model and the bottom panel illustrates the result with the freely-jointed chain model. Insets show the results for the second bond formed in each trial and better illustrate the force fluctuations by expanding the time axis. The magnitude of the force fluctuations with the stiffer rope was apparently larger than for the freely-jointed chain, but the average force on the bond was similar. The theoretical result was interesting because, if such fluctuations occur, they would result in transient peak forces larger than the mean calculated from the average bond angle and shear force. Forces experienced by the bonding pocket have an effect on molecular conformation and function [25]. The result suggested tether properties can be transmitted to the binding pocket and may influence function. Also, many bonds formed and dissociated without ever supporting a hydrodynamic load, in agreement with simulations of leukocyte rolling [43].

Although less common, several multivalent force-bearing bond events occurred. For the trial in the bottom panel in Figure 4D, this is apparent shortly before 5 s: simultaneous load bearing bonds supported a lower peak force. Video S3 demonstrates how the sphere can arrive at such a configuration: multiple force-bearing bonds also occurred with the 10 µm-diameter sphere at 7.920 s. Bonds near the edge of the contact patch can also become stressed due to stochastic excursion in the sphere's position: brief loads are apparent in the bottom panel of Figure 4D, such as at 8.972 s. Video S2 illustrates how this is possible. Bonds shorter than the deduced unstrained bond length supported no force and could form freely. While the sphere paused, it was possible for additional bonds to form in the contact patch. Although the bonds must form while unstressed, the sphere could still undergo small diffusive fluctuations in position to stress them.

### Investigation of bond loading

Mechanical loading history can be an important factor governing bond lifetimes [44]. The molecular force loading history was explicitly investigated. A simplifying assumption was made in the model: bonds shorter than the deduced unstrained bond length supported no force. As the bonds extended further, there was a step in the force/length relationship. The assumption amounted to a step to 56 pN as the length extended past the unstressed molecular contour length of 92 nm, as calculated from freely-jointed chain model parameters [21], and increased continuously thereafter. The force/length relationship is presented in detail in Figure S1.

**Figure 5:**
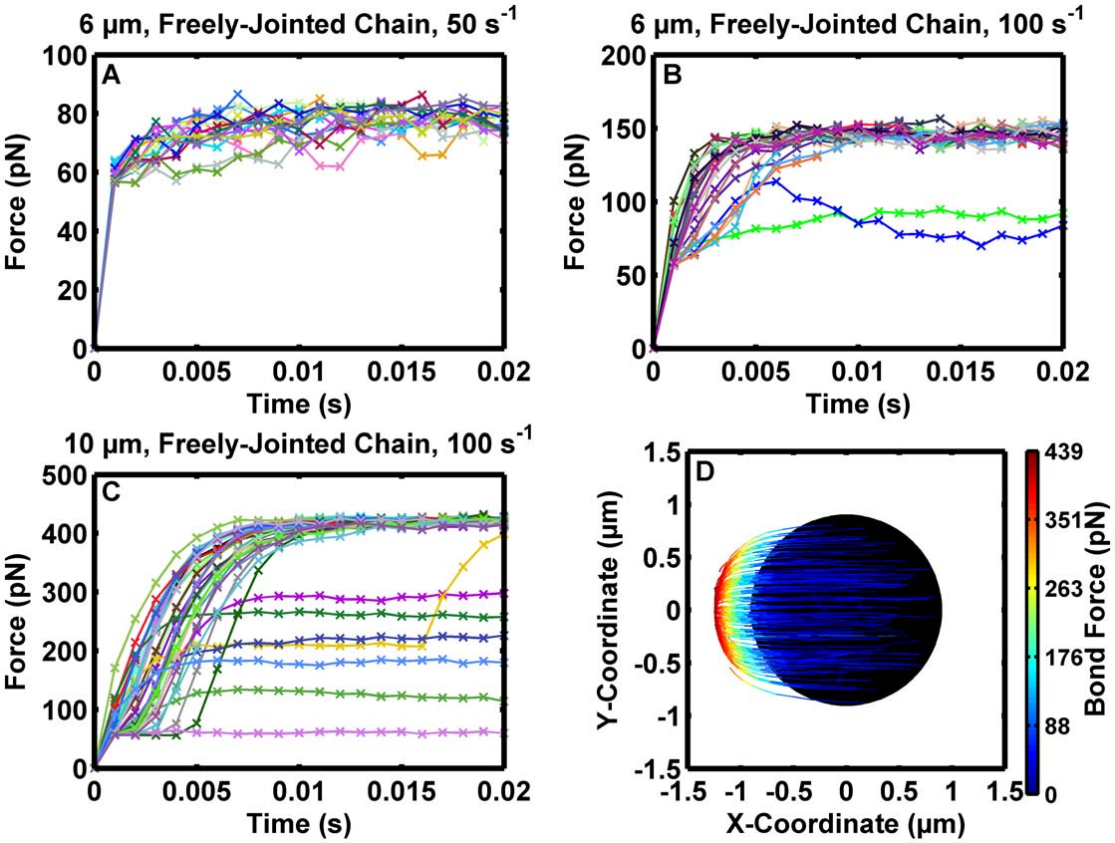
Loading patterns demonstrate how bond forces influence sphere motion. Reactive spheres were simulated with n_L_° = 100 sites/µm^2^, n_R_° = 50 sites/µm^2^, association kinetics governed by (1), and dissociation kinetics governed by (3). Note that the step function in the freely-jointed chain model, (6), resulted in a step from zero force to 56 pN as the tether extended past 92 nm, and then force continued to increase continuously. (A) A sample of bond loading data for 6 µm-diameter spheres with a 50 s^−1^ wall shear rate. (B) Results from a simulation for 6 µm-diameter spheres with a 100 s^−1^ wall shear rate. Note that bonds were aligned to their respective initial loading points in the figure, so the total instantaneous force exerted by the concurrent bonds, shown by the green and blue tracings, on the sphere cannot be calculated by summing the two values at the same time point on the plot. (C) Bond loading results from a 10 µm-diameter sphere at a 100 s^−1^ wall shear rate. Of the three cases, the larger sphere had the most bond loading events and also was the most likely to form simultaneous hydrodynamic force bearing bonds. (D) Loading trajectories for single, force-bearing bonds. Data were compiled from simulation runs for 10 µm-diameter spheres with a 100 s^−1^ wall shear rate. Individual tracings represent individual bond events, with position coordinates representing the position of the bond tether point on the sphere relative to the center, projected onto the XY plane. The color depicts the force on the bond. The black circle illustrates the expected contact patch for unstressed bonds when the sphere touches the wall to within the limits of the assumed roughness. Single bonds only supported minimal force initially, evident in the first 5 ms of loading in (C), but could only cause the sphere to wobble once the tether point exited the contact patch, apparent in (D).

Bond loading results from different simulations are presented in Figures 5A–C. Bonds were temporally aligned so loading would occur at 0.001 s. The larger, 10 µm-diameter spheres had a larger area within a molecular contour length of the surface, tended to form the most bonds, and exhibited the most frequent multiple bond formation events. Figures 5A,B illustrate the result for a 6 µm-diameter sphere interacting with a 50 s^−1^ and 100 s^−1^ wall shear rate, respectively. Although the peak load was about double in the latter case, the increased shear did not have as much of an effect on the loading rate. Also, there was a multivalent bonding event apparent with the blue and green tracings, which explains why they were not individually loaded to the full force required to restrain the sphere against hydrodynamic drag. Figure 5C illustrates the result for a 10 µm-diameter sphere interacting with a 100 s^−1^ wall shear rate, which also has a much larger contact area. Note that aside from many more binding events and more multiple loading events, some of the force tracings exhibit a prominent inflection point between 0.001 s and 0.004 s. This feature is also present, although less pronounced, in Figure 5B.

Figure 5D demonstrates bonds only become loaded and exert lateral motion on the sphere towards a mechanical equilibrium point once they leave the contact patch. Results from 10 µm-diameter spheres interacting with a 100 s^−1^ wall shear rate were screened for singly-loaded bonds and the force was plotted in relation to the receptor attachment point relative to the sphere's center. The result explains the inflection-like point in Figure 5C sometimes apparent for up to the first 5 ms of bond loading. Single bonds can become stretched just until the point they exert a restoring force, 56 pN, in the contact patch as their anchor point on the sphere rotates into the upstream hemisphere. Bond loading increases rapidly once the anchor point on the sphere rotates out of the contact patch and can then induce motion perpendicular to the flow direction, wobble.

**Figure 6:**
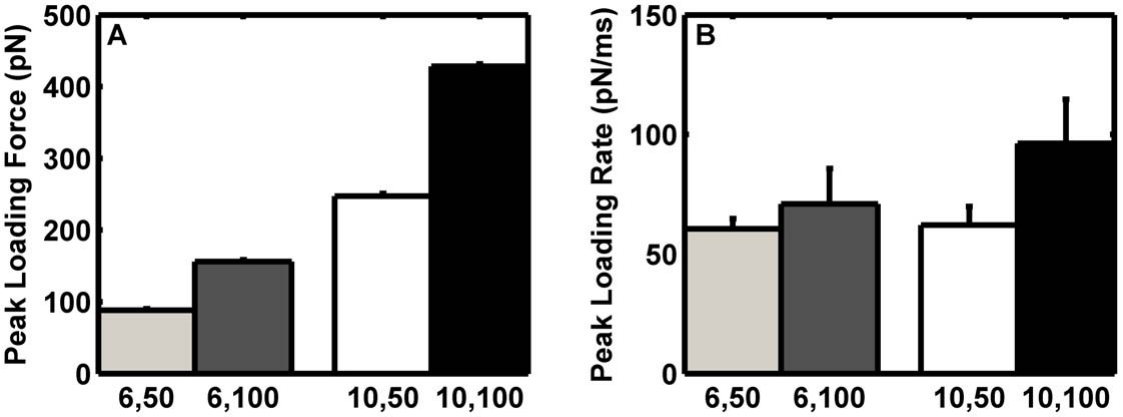
Peak loading characteristics for single bonds. Simulation results for 6 µm-diameter spheres with a 50 s^−1^ (grey) and 100 s^−1^ (dark grey) wall shear rate and for 10 µm-diameter spheres with a 50 s^−1^ (white) and 100 s^−1^ (black) wall shear rate were screened for single bond loading events. (A) Statistical compilation of mean peak single bond loading forces. The error bar depicts the standard deviation. (B) Statistical compilation of peak loading rates. The error bar depicts the standard deviation.

Figure 6 demonstrates increasing shear has a small effect on mechanical equilibrium during a pause event, where molecular bonds restrain motion of the sphere against hydrodynamic forces. The time to achieve the equilibrium configuration is slightly increased with increased shear. Singly-loaded binding events were pooled from simulation runs for 6 µm-diameter spheres with wall shear rate of 50 s^−1^, 6 µm-diameter spheres at 100 s^−1^, 10 µm-diameter spheres at 50 s^−1^, and 10 µm-diameter spheres at 100 s^−1^. As expected, the mean peak loading force increased with wall shear rate and was 88, 156, 247, and 428 pN, respectively. Note that these values correspond to the maximum of the force fluctuations for single bonds as observed in Figure 4D. It is worth noting these forces would cause dissociation of the adhesion molecule from the cytoskeleton in leukocytes and therefore decrease force on the bond [45],[46]. The peak bond force did not exhibit a strictly linear dependence on shear. The freely-jointed chain tether distensibility allowed the tethers supporting the bond to extend slightly and the lever arm angle to decrease as increased shear force increased the biomolecular distension. In all conditions, the standard deviation of the peak bond force was small: less than 4 pN. Increasing the wall shear rate had a small effect on the mean peak single bond loading rates. They were 61, 71, 62, and 96 pN/ms, respectively.

### Comparison with experiment

Having demonstrated the simulation qualitatively recreated motions observed for non-interacting spheres and also recreated the discrete, transient stops observed for interacting spheres, the next important consideration was whether the results matched detailed motion patterns observed experimentally. The site density, measured bond force-response characteristics, sampling rate, and diameter were chosen to match the previous investigation [23]. Given the alternative bond lifetime models and parameter discrepancies in the literature, several alternative dissociation models and rates were selected to test whether they might give a match to experiment. A brief comparison of alternative models of bond dissociation for P-selectin/PSGL-1 is shown in Figure S2. Two methods were employed to judge the quality of the match between the simulation and experiment. In the first, a video sample was obtained from Dr. Eric Y. H. Park. Simulation results were screened by eye to identify a period with similar qualitative behavior to a small experimental tracking data set. A detailed comparison of the motion was made. Secondly, velocity tracings from simulated microbeads were analyzed using a pause time analysis method to deduce an experimentally apparent, effective dissociation constant. Complete velocity results from the simulations employing parameters derived from the experimental study [23] are presented in Figure S3.

**Figure 7:**
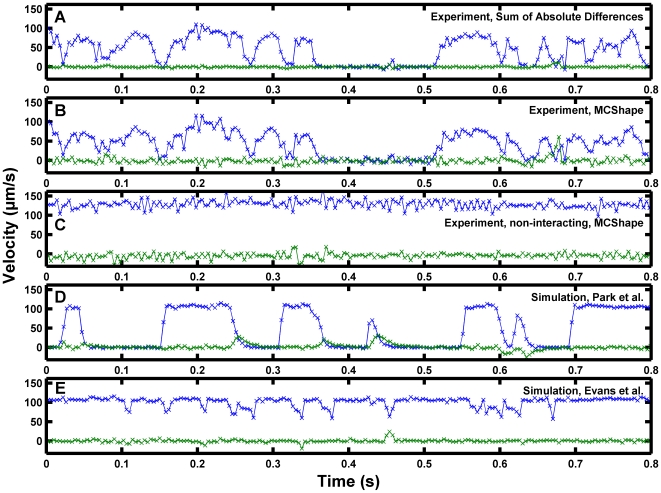
High temporal resolution comparison of simulation results to the data of Park et al. [**23**] **.** Experiments and simulations were performed with S = 50 s^−1^, R = 4.9 µm, n_L_° = 90 sites/µm^2^, and n_R_° = 95 sites/µm^2^. The sampled flow-direction velocity (V_S,X_, blue) and the sampled perpendicular velocity (V_S,Y_, green). (A) Results for an experimental microbead using the original sum-of-absolute-differences tracking algorithm. (B) The same experimental microbead was tracked using the centroid-based MCShape algorithm. (C) Tracking results using MCShape for an apparently non-interacting experimental microbead in the same video segment. (D) Simulation results using the Bell slip bond model, (3), dissociation parameters from Park et al. [23]. The comparison demonstrates the model recreates microbead motions well to a first approximation. (E) Simulation results using catch-slip model, (4), dissociation parameters from the discussion of the biomembrane force probe results by Evans et al. [22]. The results demonstrate that if the parameters discussed by Evans et al. [22] are true measures of monomeric bond dissociation under force, they would be difficult to detect by a pause time analysis of flow cell assay data.

Differences between the experimental and simulated motion patterns that suggested a refined interpretation of the experimental data needed to be made. Figures 7A,B present experimental data from the same microbead tracked by the two different methods. Figure 7A presents experimental tracking data from the previously published analysis [23] using a sum-of-absolute-differences algorithm. Figure 7B presents experimental results using a centroid tracking algorithm with intensity threshold segmentation, **MCShape**. The additional comparison set facilitated interpretation of the experimental results by establishing confidence that the velocity waveform characteristics were not noise artifacts resulting from the tracking method. The blue tracings show V_S,X_ and the green tracings show V_S,Y_. Figure 7C presents experimental data from an apparently non-interacting particle for reference purposes. Figure 7C illustrates the magnitude of the noise that can be expected from the algorithm employed in Figure 7B. Although some noise may be present in the velocity signal in Figure 7B, there is clearly a long pause beginning at roughly 0.35 s. Additional, briefer pauses are apparent with both tracking methods in Figures 7A,B. Figure 7D illustrates a selected portion of simulation results using the Bell dissociation model parameters reported for microbeads [23], sampled at 250 fps. The reduction in simulation sampling rate from 1,000 fps to 250 fps reduced the fluctuations in the sampled velocity due to diffusion. The simulation qualitatively recreated the starting and stopping events observed in the experiment, although there were fewer very short pauses in the simulation results than observed experimentally. The deceleration to a near-zero V_S,X_ agreed with experiment well, and the particle took several frames to slow in both cases. The simulation missed the lagging component in the acceleration that was apparent in the experimental data. A detectable lag period required for particle acceleration was observed previously with detachment from ligand-presenting accumulation strips [47]. There were several brief deceleration events in the experimental tracking results in Figures 7A,B that were larger than the noise in Figure 7C but missing from the simulation results in Figure 7D. The disagreement suggested an important component to the experimental physics was missed in the analysis and therefore not included in the simulation.

Pause time statistics calculated from simulated sphere motion matched experimental results. The statistical point estimate k_off_, an indicator of the dissociation rate for individual molecules or molecular clusters loaded with force, was calculated as described previously [48]. Inputting the Bell model molecular parameters experimentally measured for microbeads into the simulation [23], an apparent k_off_ was obtained from the simulated velocities that matched the statistical point estimate to within 8% (Table 2).

**Table 2:** Summary statistics for selected simulation conditions.

Cluster Valence	k_on_	Statistical Point Estimate k_off_ s^-1^	n_R_° Cluster Density ×95 sites/µm2	n_L_° Cluster Density ×90 sites/µm2	koff s^−1^	Optimal Mixed Poisson Process	Short Skip Distance µm	Long Skip Distance µm	Medium Skip Distance µm
**Dissociation Parameters and Model: Park et al. (3)**									
1	(1)	12.6	1×	1×	12.0	2	0.69	12.89	
1	(2)	12.6	1×	1×	11.6	2	0.58	15.23	
**Dissociation Parameters and Model: Marshall et al., Catch-slip (4)**									
1	(1)	100	1×	1/2×	51.7	2	1.81	46.17	
1	(2)	100	1×	1/2×	66.1	2	3.24	43.84	
1	(1)	100	1×	2/3×	64.6	2	3.24	34.71	
1	(2)	100	1×	2/3×	70.3	2	0.30	33.15	
1	(1)	100	1×	1×	55.5	2	0.40	21.45	
1	(2)	100	1×	1×	72.4	2	1.12	21.18	
1	(1)	100	1×	2×	59.3	2	0.93	8.80	
1	(2)	100	1×	2×	53.6	2	1.01	9.83	
1	(1)	100	1×	4×	41.2	2	0.44	3.51	
1	(2)	100	1×	4×	37.1	3	0.22	5.32	0.86
2	(1)	20.5	1×	1×	22.0	2	0.85	13.38	
2	(2)	20.5	1×	1×	21.3	2	0.83	12.27	
1	(1)	100	2×	1×	60.3	2	0.73	9.34	
1	(2)	100	2×	1×	55.5	2	0.89	9.64	
3	(1)	10.0	1/3×	1/3×	10.4	2	2.02	118.45	
1	(2)	10.0	1/3×	1/3×	14.5	2	0.65	82.56	
**Dissociation Parameters and Model: Marshall et al., Slip Only (3)**									
1	(1)	100	1×	1/2×	62.7	2	2.60	37.78	
1	(2)	100	1×	1/2×	61.8	2	9.62	36.63	
1	(1)	100	1×	2/3×	69.1	2	1.47	29.48	
1	(2)	100	1×	2/3×	55.1	2	1.18	27.51	
1	(1)	100	1×	1×	56.0	2	1.03	20.46	
1	(2)	100	1×	1×	56.0	2	1.08	23.19	
1	(1)	100	1×	2×	59.3	2	0.79	9.48	
1	(2)	100	1×	2×	53.6	2	1.01	9.83	
1	(1)	100	1×	4×	38.7	3	0.16	5.34	0.73
1	(2)	100	1×	4×	29.3	3	0.13	7.95	0.65

### Model predictions

There have been many measurements of selectin kinetics and mechanical responses. The reported measurements vary by orders of magnitude. Analyses have incorporated receptor multivalency [22],[48],[49] as well as cellular deformability and microvillus elongation [23], [48]–[51] as possible reasons for the discrepancies. The experimental result of Evans et al. [22] represents a monomeric bond formation case. Simulation results assuming parameters estimated from the discussion [22] are presented in Figure 7E. The result demonstrates monomeric bonds would not result in pauses, at least for 10 µm-diameter spheres. Some bond events might not even be detectable above noise. Comprehensive results from the simulations employing the monomeric parameters [22] are presented in Figure S5. The results suggest the transient deceleration events in Figures 7A,B that did not pause the sphere could be low-valency bond formation events.

A conclusion of previous studies has been that dimerization plays a significant role in measured cellular bond lifetimes [48],[49]. We refer to multivalent molecular groupings that form bonds as a unit and evenly distribute a force as clusters. Reliability theory rules governing cluster dissociation, similar to those employed in previous analyses of bond lifetime [52], were added into the present model. The goal was to investigate whether bond clusters could account for the observed discrepancies in the flow cell microbead pause kinetics with the parameters measured by molecular force spectroscopy techniques. Clusters were assumed to form at the same rate as monomers. This assumption facilitated the interpretation of the motion statistics, although dimerization has been reported to result in a two-fold enhancement on bond formation rates, as assessed by detected pause events [53]. The summary statistics for a variety of simulation conditions are compiled in Table 2. A more comprehensive compilation of results is available in Table S1.

Reliability theory was used to create a dimeric grouping of the catch-slip parameters obtained from experiments with dimeric P-selectin/PSGL-1 interactions [5],[32], which might physically correspond to tetrameric bond clusters. The statistical point estimate obtained for the dimeric dimers indicated dissociation kinetics still faster than observed experimentally. The observed k_off_ calculated from the simulation also closely matched the statistical point estimate. Trimeric groupings of the catch-slip dimers [5], which might correspond to hexameric clusters, produced dissociation kinetics slightly slower than experiment (Table 2). Simulation results suggested the flow cell experiment [23] was primarily detecting dimeric to trimeric groupings of dimers measured in the force spectroscopy experiment [5]. The cluster had to be increased to 3× dimers for the observed k_off_ to approach that reported by Park et al. [23] (Table 2). Interestingly, membrane P-selectin has been reported to form noncovalent hexamers under some isolation conditions [37]. Membrane PSGL-1 has also been observed to form rosettes [36]. A thorough analysis optimizing cluster size distributions to match bond lifetime data has been previously performed for cellular systems [48],[49]. Therefore, subsequent analysis investigated what might be expected from an experimental microbead flow system similar to the previous study [23], except with the molecules immobilized in a dimeric configuration.

Fluctuations in V_S,Y_, wobble, might also contain information about biomolecular tether formation events. The simulation results in Figure 7D demonstrate brief increases in the magnitude of V_S,Y_ at the same time V_S,X_ is observed to decrease. The wobble was not readily apparent in the experimental velocity results. The tracking results in Figure 7A exhibited little variation in V_S,Y_. The algorithm employed in Figure 7B exhibited random variations in V_S,Y_ that appeared to be noise. There is one event just before 0.7 s that might correspond to a real wobble. The movies were taken with a 20× objective and the movie quality would be improved with current technology. It is possible better resolution will detect real wobble.

**Figure 8:**
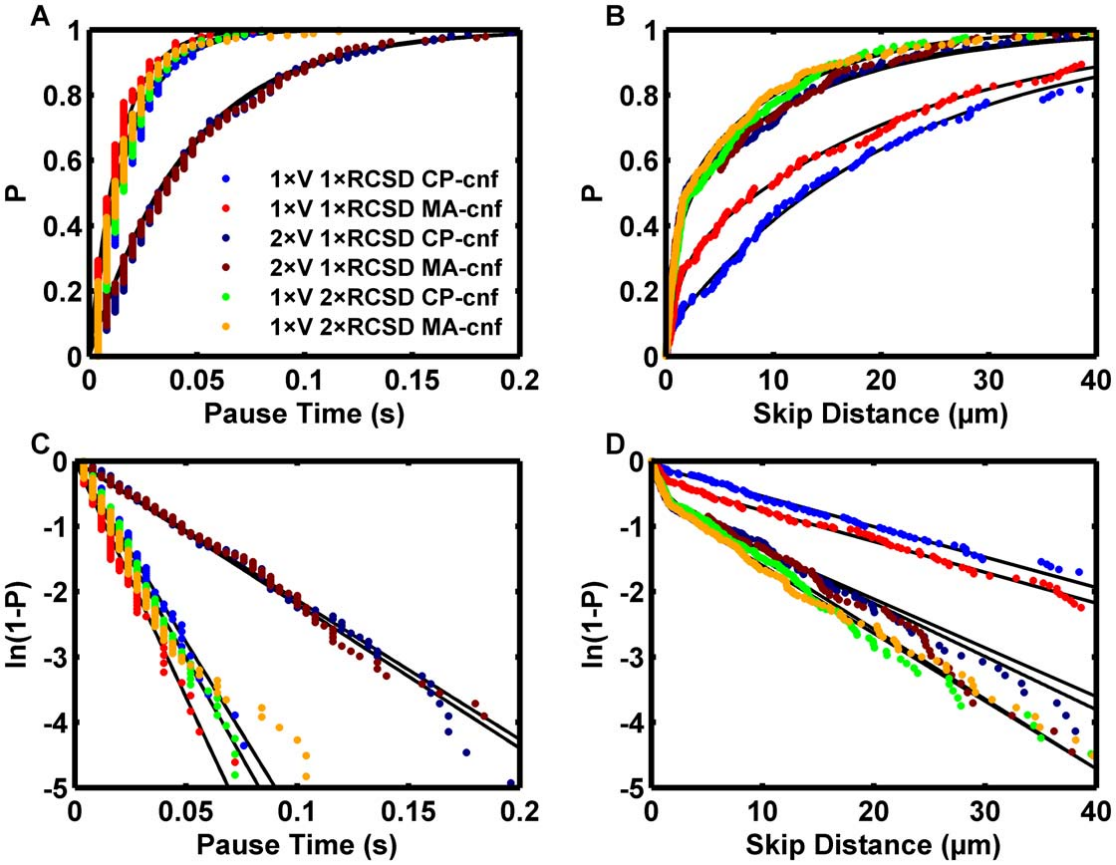
Pauses primarily affected by clustering but skips primarily sensitive to available binding pockets. Reactive spheres were simulated with S = 50 s^−1^, R = 4.9 µm, n_L_° = 90 sites/µm^2^, 1×n_R_° = 95 sites/µm^2^, conditions similar to the experiment of Park et al. [23]. Association kinetics were governed by (1) or (2). The catch-slip dissociation model, (4), was used employing parameters regressed from the flow-cell data of Marshall et al. [5],[32] for dimeric interactions. Increased valency, V, was achieved for each receptor cluster site by using reliability theory rules [52] to create load-sharing molecular clusters. Therefore, 2×V might physically correspond to a tetrameric bond cluster. The black lines show the fit parameters reported in Table S1 and the dots show data points from the simulation. The percentile, “P,” indicates the uniform order statistic median. Each data set was pooled from three 10 s simulation runs. Blue: single valence receptor clusters with contact patch confinement. Red: single valence receptor clusters with molecular area confinement. Dark blue: double-valence receptor clusters with contact patch confinement. Maroon: double-valence receptor clusters with molecular area confinement. Green: single valence receptor clusters but with double the receptor cluster site density and contact patch confinement. Gold: single valence receptor clusters but with double the receptor cluster site density and molecular area confinement. (A) Non-transformed pause time data. (B) Non-transformed skip distance data. (C) Linear transform for Poisson-distributed pause times. (D) Linear transform for Poisson-distributed skip distances shows two distinct regions.

Higher site densities do not mediate extended pauses nearly as well as when the receptors and ligands are packaged into molecular clusters, as shown in Figure 8. The catch-slip parameters regressed from the dimeric flow cell data [5],[32] were used for further analysis. They represented a minimum achievable valency configuration for experiments employing wild-type PSGL-1 and were able to detectably pause the sphere. Comprehensive velocity results with these parameters are shown in Figure S4. The effect of receptor distribution was tested by increasing cluster valency or alternatively increasing the number of receptor clusters. The apparent k_off_ was estimated from the slopes of the black lines in Figure 8C. Some quantization was apparent in the pause time values due to the brevity of the pause relative to the sampling rate. The receptor cluster site density basis was 95 sites/µm^2^. The results with 1× and 2× receptor cluster density were similar for an assumed cluster valence of one, suggesting multiple hydrodynamic load-bearing attachment points could not form efficiently. It was necessary to package receptors and ligands into molecular clusters to effectively extend pause times.

Recent observations suggest that skip distances are an important measure of biomolecular binding efficiency [39]. An analysis of how far the modeled sphere traveled between the pause events was performed. Single-component Poisson models could not reconcile the initial steep slope and shallower response phase at longer skip distances apparent in Figure 8B. A logarithmic transformation was employed, as shown in Figure 8D. Two linear segments were apparent in the transformed data, suggesting a statistical model blending multiple Poisson processes might match well. The mixed Poisson process model was tested:
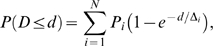
(7)where
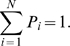
(8)


Here, d is the skip distance, the P_i_'s are the probability of one of the N Poisson processes, and the Δ_i_'s are the respective rate parameters with dimensions of distance. Parameter estimates were derived using nonlinear regression in MATLAB. The results for a two-component Poisson process are plotted as black solid lines in Figures 8B,D. The regression fit the data well. A physical explanation for the high probability of short skip distances relative to a single-component model would be the existence of pre-existing bonds in the contact patch. When the hydrodynamic load-bearing bond breaks, the sphere could only perform a long skip if no pre-existing bonds were present in the contact patch to catch the sphere. Indeed, the short-skip distance derived from the regression was on the order of the size of the patch where molecules on the sphere could contact the surface, one micrometer (Table 2). Figures 8B,D illustrate, as expected, the skip distance was most effectively reduced by increasing the density of receptors on the surface. Surprisingly, doubling the valency with a constant cluster density was almost as effective at reducing the skip distance as doubling the site density with constant valency, despite a constant association rate. The result demonstrates dissociation kinetics can influence measures of bond formation. The result also reinforced the conclusion that functionally effective molecular interactions require clustering.

**Figure 9:**
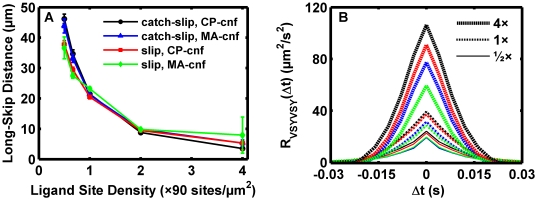
Long-skip distances and wobble differentially reflect molecular activity. Reactive spheres were simulated with S = 50 s^−1^, R = 4.9 µm, 1×n_L_° = 90 sites/µm^2^, and n_R_° = 95 sites/µm^2^. Association kinetics were governed by (1) or (2). Catch-slip parameters regressed from Marshall et al. [5],[32] were used in the catch-slip model, (4), or the just the high-impedance pathway parameters were entered into the slip model, (3). (A) The long-skip distance calculated from the two-parameter Poisson regression at a variety of ligand concentrations. Black circles: the catch-slip model was assumed with contact patch confinement. Blue triangles: the catch-slip model was assumed with molecular area confinement. Red squares: the slip model was assumed with contact patch confinement. Green diamonds: the slip model was assumed with molecular area confinement. Error bars show the 95% confidence interval estimate from the nonlinear regression. Surprisingly, the dissociation model had a moderate effect on the skip distance at low site densities but the confinement model had little effect. (B) The sample-normalized autocorrelation of the velocity component perpendicular to the flow direction (wobble velocity autocorrelation) was calculated and averaged together for the three simulated beads at each condition. The colors indicate the same reaction assumptions as in the previous graph. Heavy dashed lines: results from 4×90 sites/µm^2^ ligand density. Intermediate dot-dashed lines: results from 1×90 sites/µm^2^ ligand density. Light solid lines: results from ½×90 sites/µm^2^ ligand density. Colors represent the same cases as in (A). The confinement model had the largest effect on the wobble autocorrelation.

To investigate the effects of the catch component in catch-slip bond formation, simulations were also run assuming the high-impedance pathway parameters derived from the dimeric flow cell study [5],[32], entered as a Bell slip model. The results are indicated as “slip only” in Table 2. If catch-slip bonds were present, it was postulated they might result in a decreased sensitivity of the short-skip fraction to the amount of ligand available. A test of the functional impact of reaction enhancement due to confinement was included in the analysis by comparing the results with different receptor bond formation rules defined by (1,2). Reactions enhanced by molecular area confinement might be expected to result in smaller short-skip distances than reactions exhibiting contact patch confinement. Instead, a distinguishable effect of the confinement model on the short-skip measurements was not observed. This may be due to the smaller sample size at lower ligand densities. However, a robust indicator of biomolecular activity should have a detectable pattern even with the smallest sample size of 24 events. Instead, an emerging trend in the long-skip data was apparent in Figure 9A. Surprisingly, the long skip distances clustered better according to the functional form of the off-rate than the on-rate at low ligand densities. At the lowest ligand density, a roughly 25% reduction in the long skip distance was observed with the “slip only” bonds. The results emphasize the difference between molecular tether formation and pausing. The differences due to the assumed off-rate model suggest that when interaction is mediated by a small number of bonds, the dissociation kinetics influence the ability to initiate a pause. A reasonable explanation for the observed decrease in the long-skip distance with the removal of the catch component is that the catch component increased the dissociation rate of transient molecular tethers before they could rotate out of the contact patch, become stressed, and effectively pause the microsphere.

The difficulty in making deductions about molecular confinement from skip distances with the selected particle size and dissociation parameters suggested another experimental metric was necessary. Therefore, we investigated whether motion perpendicular to the flow direction, wobble, carried information regarding two-dimensional formation kinetics. The probability distribution function for V_S,Y_ was identical at a given ligand concentration, regardless of the assumption of the functional form of the molecular formation or dissociation rate (data not shown). The identical probability distribution functions for V_S,Y_ suggested diffusive motion obscured the analysis. The persistence of a wobble was investigated using autocorrelation. It was reasoned that bond-directed rather than diffusive-directed motion should correlate over short time intervals as a bond became stressed. The autocorrelation of V_S,Y_ yielded informative results and is presented in Figure 9B. At low ligand concentrations, the wobble autocorrelation grouped very well by the assumed confinement model. Confinement-sensitive biomolecules wobbled the sphere less, as their tether anchor points on the sphere were more likely to be more proximal to the center of the sphere's planar projection. As ligand concentration increased, the dissociation model also played a role, although smaller, in the particle wobble.

**Figure 10:**
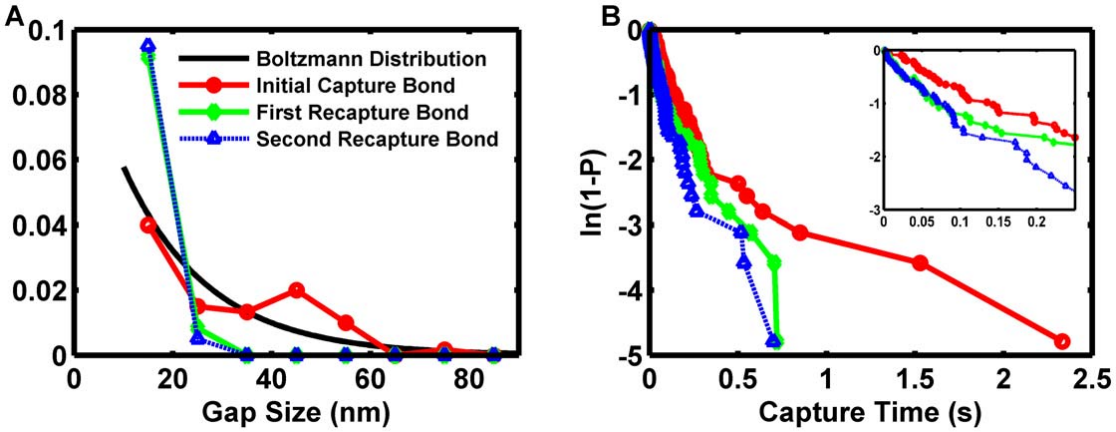
Molecular capture spans the gap and enhances recapture events. The simulation results from Figure 9 were pooled to obtain 60 events for each capture bond. (A) An analysis was conducted to investigate the separation of the sphere from the surface when bonds form in the absence of pre-existing bonds. First capture bonds formed efficiently across the range of gap sizes. Solid black line: equilibrium distribution for the gap size. Red circles: distribution of separation distances when the first bond formation event occurs. Green diamonds: distribution of separation distances observed when the first recapture, e.g. second capture, bond forms. Blue dashed line with triangles: distribution of separation distances when the second recapture, e.g. third capture, bond forms. (B) Once the sphere captured to the surface once, the first and second recapture bonds formed more quickly. The characteristic binding times describing the first linear segment (inset) for the initial capture bond, the first recapture bond, and the second recapture bond were 0.143 s, 0.081 s, and 0.085 s, respectively.

The role of surface separation in initial capture and recapture events was investigated. The results from the simulations investigated in Figure 9 were pooled. A capture bond was defined as a bond that formed when there were no existing bonds in the previous time step. The distribution of gap sizes during the first capture bond and subsequent recapture bond events is presented in Figure 10A. The distribution was pooled from 60 simulations, and the result for the first capture bond agreed relatively well with the Boltzmann distribution governing the separation at equilibrium. Therefore, at the site densities employed, the PSGL-1/P-selectin pair effectively reached across the 90 nm gap to mediate initial bond formation. There was not a detectable requirement for the sphere to undergo a thermal excursion closer than the molecular contour length to form a bond. Bond recapture events, which would roughly correspond to “long skips” in Figure 9B, were observed to occur at smaller separation distances. The result suggested that subsequent bond formation events should occur more quickly than the initial because more receptors would be within an unstressed molecular contour length of the wall, as suggested by (1,2). In Figure 10B, the time until the first capture event is plotted as well as the interval between bond breakage and recapture. Indeed, recapture occurred more quickly than initial capture.

### Relative motion effects

The lateral motion of the stationary and moving surface relative to each other, as observed in the rolling of leukocytes, can affect the rate of reaction in some situations and merits specific consideration. A departure from the previous analysis of lateral relative motion [54] would need to be implemented for the process investigated presently for two reasons. First, a small number of bonds with significant changes in their relative number can be observed in Figures 4A–C, demonstrating the present process is not near steady-state and is therefore inconsistent with the assumptions of the previous analytical model [54]. Secondly, the appropriate diffusivity model for molecular binding pockets firmly attached to an immobile anchor point on a surface by a tether is qualitatively different than one in which the tether attachment point is also free to diffuse in a membrane. To better elucidate these points, consider the general equation for convective and diffusive transport:
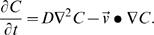
(9)


For initial biomolecular bond formation, or for the case where there are significant fluctuations in the free ligand density due to the stochastic nature of bond formation, the time derivative of the concentration will not be zero as assumed in the previous relative motion analysis [54]. The transition from no bonds to at least one bond, as during initial tethering, is not a steady-state process, and it will be desired to accurately capture this step. Secondly, it is not intuitively clear what the diffusivity constant in (9) represents if the reactive end groups are free to diffuse about a tether point but the surface attachment point is not mobile in a membrane. As the sphere rotates, receptors that have formed bonds or are engaged in encounter complexes must dissociate for the sphere to move forward because the receptor attachment points are fixed on the sphere's surface. A more detailed treatment is presented in Protocol S2. Therefore, relative motion cannot enhance bond formation by lateral transport effects when the receptors are immobilized to a point on the surface of the sphere and ligands are also immobilized on a surface, as in the present case.

It is possible that relative motion might decrease bond formation. If the sphere moves fast enough such that the receptor and ligand binding pocket, once they happen upon a suitable encounter, cannot complete reorientation of residues in the binding pocket to complete the bond, no bonds can form. The requirement for bond formation introduced by the consideration of relative motion is:
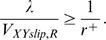
(10)


This portion of the motion analysis is similar to previous studies [54],[55]. V_XYslip,R_ is the slip velocity a receptor on the sphere relative to the surface, and V_XYslip,R_ is less than the sphere's velocity due to the rotation of the sphere. The reaction rate, r^+^, is similar to previously described intrinsic reaction rates [1],[54],[55], except an additional transport step can be removed and made explicit, as discussed in Protocol S2 and shown in Figure S6. The intrinsic bond formation rate should be very fast, and the timescale has been projected to be around 10 µs or less from simulations [54]. The quantity on the left in (10) should be around 1,000 µs for the shear rates employed here. A decrease in reaction due to relative motion would not be expected for the shear rates employed.

## Discussion

Several key findings were made in the present investigation. The first two of these were especially apparent through a detailed analysis of sphere's motions in the simulation. First, the grouping of molecules into load-sharing clusters is critical for function. Single bond formation events cannot pause the sphere at the wall shear rates investigated because a single receptor-ligand bond cannot withstand the force. Secondly, the wobble autocorrelation may serve as an indicator of confinement enhancement in the molecular formation kinetics. Finally, it was also observed experimentally measured P-selectin kinetics and densities are able to effectively capture a particle as long as the particle is within a molecular contour length of the surface. Furthermore, recapture is enhanced by the proximity to the wall.

The simulation method presented here differs in several important fundamental ways from previous computational models of adhesive interactions in flow. A theoretical framework for modeling the vertical and lateral diffusion of microspheres under flow was previously developed [56], but the previous investigation did not incorporate biomolecular bond formation. The implementation presented here also adds rotational motion and rotational diffusion, since they were needed to track the position of individual receptors and molecular tether attachment points. Previous work developing adhesive dynamics simulations provided an invaluable reference and a presentation of many of the components of the physics employed [57]. The inclusion of thermal motion enabled the investigation of the effects of surface separation on capture and direct comparison to experiment. Many adhesive dynamics simulations aim to discern how more complex factors integrate to influence cellular rolling behaviors (for example: [32],[58],[59]). The model presented here did not incorporate cellular factors to try to integrate all of the influences on leukocyte rolling. Rather, the present goal was to answer questions about biomolecular reaction. The effects of surface separation and molecular characteristics governing bond formation on two-dimensional biomolecular kinetics are fundamental questions of biomolecular function. Although the present investigation focuses on the selectins, which are very important to a variety of vascular homing processes, the methodology and results may be applicable to additional classes of two-dimensional bimolecular interactions.

Good simulated pausing and skipping results were achieved using physiologic site densities [14] and recently published reaction rate data [60]. Our initial attempt to model rolling behavior using the previously published k_f_ value of 1.7 µm^2^/s [20] did not result in rolling: the sphere formed too many bonds to move. The sphere exhibited good rolling behavior when we employed a k_f_ of 4.8×10^−4^ µm^2^/s, which was extrapolated from the <A_C_ k_f_^o^> recently reported [60]. It is very likely the estimation of the site density was improved in the more recent study. It is also of note that the two different formation rate estimates come from two different measurement methods: the biomembrane force probe and the laser trap. The configuration of the two experiments was different. With the biomembrane force probe study, the two surfaces were held some small distance apart, whereas with the laser trap the two surfaces were pushed together. It is possible an increased confinement of the reactive groups increased the bond formation rate in the measurement with the laser trap. However, it does not seem likely confinement would account for a 3,500-fold increase in reaction rate.

The simulation method might be employed in the future to investigate the influence of bond formation rates and contour lengths on pausing and skipping behaviors, given the observed sensitivity to the bond formation rate, k_f_, and vertical transport. A state diagram of their influence may be informative [34]. A direct comparison individually trading each molecular parameter measured for L-selectin and P-selectin should better elucidate the impact of their molecular adaptations in capture and rolling. Although only one model of confinement effects was investigated here, the molecular area confinement model described by (2), the simulation can be employed to investigate other functional relations describing the confinement effect.

The simplified assumptions of the forces governing z-motion in the simulation may also miss interesting behaviors. An interesting potential result of adding a repulsive layer is that a bond might not simply drive the sphere to the wall as in the present work. Subsequent binding events might ratchet the sphere further into the layer due to the highly damped nature of the vertical diffusion. The recapture time could decrease much more substantially with subsequent binding events. Such an effect might effectively couple an increase in shear rate with an increased force pushing the sphere into the repulsive layer, enhancing recapture with increasing shear. Experimental progress has been made to analyze the near-wall vertical motion of microbeads in low ionic strength solutions using total internal reflection microscopy (TIRM) [61]. Future experimental efforts might utilize TIRM methods to analyze more physiologic conditions with higher ionic strength buffers and protein coatings. In silico and in vitro glycocalyx analogues could be constructed [9].

Investigations have found an increase in apparent selectin-mediated cell and microbead capture with increasing shear [29],[39],[62],[63]. Several explanations have been proposed: increased force increases the molecular bond formation rate [39],[62], the motion of the two surfaces relative to each other increases reaction through lateral transport [54],[63], and an increase in cell flattening with increased shear may enhance tethering [49]. Here, it was discovered recapture is enhanced by vertical transport closer to the wall, independent of cell deformation [51]. The simulation results suggest an additional factor that may contribute to enhanced adhesion under flow conditions.

The lack of an enhancement in the bond formation rate due to lateral relative motion transport for the present simulation system is not in conflict with previous computational studies [54]. The physical configuration of the previous system was substantially different. Most significantly, the points where the receptors and ligands were attached to their respective surfaces were free to move in the membrane. A receptor and ligand pair could therefore remain in the contact patch if they happened to find each other as the sphere rolled. The lack of an enhanced effective bond formation rate due to lateral relative motion transport in the present analysis does appear to be in conflict with the conclusion of an experimental study employing immobilized receptors [63]. The conclusion that particle sliding, lateral transport, enhances the binding rates is consistent with the presented experimental scaling data and implies that a lateral transport mechanism governs the formation rate. However, other mechanisms that enhance the bond formation rate and scale similarly with shear and size could also account for the result. For example, inter-particle hydrodynamic interactions can influence vertical transport to the wall [47],[64]. Notably, the frequency of inter-particle interactions would increase with increasing shear. Vertical cell or bead mixing with the surface might increase with increasing shear by inter-particle interactions. Also, force increases with increasing shear, and other studies have suggested increasing force might increase the bond formation rate [39],[62].

The analysis developed in Protocol S2 suggests transport in the form of lateral sliding should not enhance formation rates when the receptors and ligands are attached to their respective surfaces by an immobile anchor point. An experimental microbead study suggested molecular bond formation rates might be force dependent [39]. This was a bold assertion given the implications for biomolecular reaction theory. The discrepancy in the conclusions between published microbead studies [39],[63] suggests the simulation developed here be employed with L-selectin parameters and coupled with experiment. It is likely vertical transport plays an even more significant role in systems with L-selectin than was observed in the present results. L-selectin has fewer repeated subdomains and is shorter than P-selectin, which should enhance the importance of vertical transport. Also, the microbeads used in these experimental studies [39],[63] were smaller than 10 µm. In Figure 3, it is apparent the smaller spheres diffuse away from the wall more frequently. Therefore, with these experimental systems [39],[63], a more significant effect of the confinement model might be observed. Directly coupling a computational methodology with experimental observations of selectin-mediated particle interactions with TIRM or total internal reflection fluorescence microscopy (TIRFM) would facilitate direct observations of vertical fluctuations and a conclusive analysis [65].

If the reaction rate enhancement in both microbead studies [39],[63] is due to the same mechanism and is truly biomolecular in nature, rather than due to transport, the rate enhancement could be interpreted as a macromolecular version of collision or transition state theory as originally developed for covalent bond formation kinetics. The energy scales involved are interesting. From Figure 3B, the modal velocity for a 6 µm-diameter bead at 50 s^−1^ is roughly 90 µm/s. At 100 s^−1^, near the peak tethering rate in the study [63], a 6 µm-diameter bead would have a modal kinetic energy equivalent to 0.5 k_B_T. The kinetic energy of the particle could be coupled to the reaction efficiency of the binding pocket through the molecular tether.

It has been suggested that apparent increases in the cellular tethering rate with shear [29] may be due to increased flattening of the cell [49]. Cell morphology is much more complicated than the rigid sphere case we have considered here. Sedimentation effects on the microvillus length scale may play a role as cellular protrusions bump into the functionalized surface [17]. Additionally, it has been illustrated here that bond dissociation properties may influence measures designed to test bond association. Another factor could be a small lift effect [66],[67]. Lift should not be important based on the wall proximity criterion. However, even nano-scale vertical displacements have functional binding consequences due to the molecular nature of the capture events.

The molecular loading rates observed in Figure 6 begin near the higher limit of the range employed in the biomembrane force probe study of dissociation pathway switching in P-selectin (0.02–40 pN/ms) [22]. It was therefore interesting that there was still an observable effect of the off-rate on the observed long-skip distance in Figure 9A, likely because the bonds were unstressed for a brief period. Interestingly, a study with (poly)ethyleneglycol linkers found thermodynamic fluctuations in the molecular tether allowed receptors to extend, bind, and then exert an attractive force between the two surfaces [68]. The assumption that molecular tether points must be brought within the molecular contour length and exert no force upon binding serves as a simplifying first approximation. Measurements of catch-slip bonding do exhibit a striking numerical relationship with previous molecular measurements of length and force. Force at the non-deformed molecular length of 92 nm was 56 pN, as calculated from the freely-jointed chain model parameters [21]. For a dimer, this force distributes as 28 pN per binding head, very close to the reported catch-slip optimum for P-selectin [5]. The peak loading rates in Figure 6 agree with those deduced for neutrophils tethering in a flow cell [29]. Simulations have shown how dissociation of a receptor from the cytoskeleton and microvillus extension can decrease the load on a tethering biomolecular complex, and it was noted clustering plays an important role in cellular bond lifetime [49]. Here, it was observed that in rigid microbead flow assays, the bond loading might be higher but clustering still can explain the discrepancy in the results between measurement methods. Indeed, comparing Figures 7C,E, it might be difficult to make observations of single-molecule bond formation events in a flow cell without a careful experimental design.

Although the present study suggests molecular confinement is not important to enhancing the function of molecular pairs mediating transient interactions, confinement has been suggested to play a role for molecular pairs that must mediate longer-lived interactions [7]. Although flow cell techniques have frequently been used to investigate interactions involving selectins, they have been applied to more classes of molecules such as antibodies, cadherins, and T-cell surface molecules [69]–[71]. The wobble autocorrelation measurement should be broadly applicable to more classes of molecular interactions than the P-selectin/PSGL-1 interaction explicitly explored here, where it might better indicate adhesive function.

The implementation of a new modeling methodology to investigate the important qualitative and quantitative characteristics of molecular systems mediating two-dimensional interactions was reported here. In addition to exploring the molecular characteristics and parameters important for other closely related adhesion systems, such as L-selectin/PSGL-1, we anticipate the computational method will be extended to entirely new molecular systems. For example, polyethylene glycol tethers have a pronounced influence on particle interactions with immobilized ligands [72]. Properties such the effective tether extension and the compressibility of the surrounding polyethylene glycol coat might be designed using computational modeling for optimized vascular binding to molecular targets. Although the present focus has been on understanding dynamic interaction, e.g. biomolecular tethers arresting particles, the technique should be entirely applicable to new classes of molecules. The investigation of confinement was inspired by a study of CD2/LFA3 interaction, an important component of extended adhesion and signaling between T-cells and antigen presenting cells [7]. The technique should therefore be broadly applicable to additional classes of inter-cellular interactions. The importance of confinement in mediating long-lived interactions has been suggested to be a result of the closely-controlled intermembrane distance, which is not fixed in dynamic adhesion. For example, the diffusion of individual receptors might be added into a discrete receptor model to watch how molecules assemble into the synapse at the interface during the extended adhesive interaction. Furthermore, the computational methods might be used to optimize the molecular properties, such as length and flexibility. Intercellular bond formation could be linked to intracellular signaling cascades and the spatial localization of signaling scaffolds. This could facilitate the design of functionally-enhanced dendritic cells for immunotherapy or re-engineering dendritic cell subpopulations to elicit a desired T-cell (usually TH_1_) differentiation pathway [73].

## Supporting Information

Protocol S1 Additional microsphere motion modeling and analysis details. An in-depth description of the model assumptions and calculations is given. The statistical analysis of the simulation results and the implications of noise in the acquisition system are discussed further.
(0.16 MB DOC)

Protocol S2 Additional lateral transport considerations. The negligible effect of lateral transport on the bond formation rate under the simulated experimental conditions is discussed further. An alternative model of encounter complex and bond formation that could be used to incorporate lateral transport effects is presented.
(0.03 MB DOC)

Table S1 Compilation of simulation results.
(0.31 MB DOC)

Figure S1 Assumed models of force extension. The employed models of molecular elongation with force are compared to experimental data and alternatives.
(0.06 MB DOC)

Figure S2 P-selectin/PSGL-1 bond lifetimes with alternative force dissociation models, parameters, and valencies. The bond dissociation rate as a function of force is shown for several published measurements of P-selectin/PSGL-1 bonds.
(0.08 MB DOC)

Figure S3 Velocities using the Bell model dissociation parameters of Park et al. Instantaneous velocity results from simulations utilizing the dissociation kinetics reported by Park et al. are presented as a function of time.
(0.84 MB DOC)

Figure S4 Velocities using the five-parameter catch-slip model dissociation parameters derived from the experiments of Marshall et al. Instantaneous velocity results from simulations utilizing the dissociation kinetics from the study by Marshall et al. are presented as a function of time.
(0.94 MB DOC)

Figure S5 Velocities using the five-parameter catch-slip model dissociation parameters of Evans et al. Instantaneous velocity results from simulations utilizing the dissociation kinetics from the study by Evans et al. are presented as a function of time.
(0.65 MB DOC)

Figure S6 A model of encounter complex formation and bond formation. A model accounting for the role of both the molecular tether and binding pocket chemistry in bond formation is presented. The model can be employed with the assumption that the receptor and ligand are each confined by an immobile tether anchor point to a separate surface.
(0.16 MB DOC)

Video S1 Movie showing a selected 1 s of interactions for the microsphere in Figure 4A.
(1.32 MB MPG)

Video S2 Movie showing a selected 1 s of interactions for the microsphere in Figure 4B.
(1.24 MB MPG)

Video S3 Movie showing a selected 1 s of interactions for the microsphere in Figure 4C.
(1.30 MB MPG)
