# Supplementary material for: Nano-motion Dynamics are Determined by Surface-Tethered Selectin Mechanokinetics and Bond Formation
Source: PLoS Comput Biol. 2009 Dec 18;5(12):e1000612. doi: 10.1371/journal.pcbi.1000612 (PMC2787012; doi:10.1371/journal.pcbi.1000612)
Supplement: Protocol S1 — Additional microsphere motion modeling and analysis details. An in-depth description of the model assumptions and calculations is given. The statistical analysis of the simulation results and the implications of noise in the acquisition system are discussed further. (0.16 MB DOC) [file pcbi.1000612.s001.doc]

**Protocol S1. Additional microsphere motion modeling and analysis details.**

**Vertical force assumptions**

The sphere was assumed not to approach the wall closer than 10 nm. The roughness of materials employed in experimental assays has been suggested to range from 25 nm to 150 nm [1-3]. Ideally, roughness can be minimized in future experimental assays by a careful selection of materials, such as glass [4]. We wished to avoid theoretical complications that might result from roughness exceeding the length of a molecular bond and leave this as an experimental concern for optimal assay design. It has been suggested van der Waals forces do not significantly influence behavior in flow cell assays [5], so they were assumed to be negligible. A repulsive steric stabilization potential could be introduced to capture the short-range effect of crushing a layer of coating lipids or proteins [6]. However, the steric stabilization layer may be significantly different depending on the method of ligand immobilization and blocking employed, so the force was neglected. It should be possible at present to make approximate quantitative predictions without the steric layer. A more precise analysis with accurate knowledge of steric layer parameters would be limited in generality. Electrostatic double-layer repulsion was neglected. The ionic strength of physiologic buffers results in a Debye length of less than 1 nm [7] and the electrostatic force would be less than the steric stabilization force [8], which was neglected. Having ruled out inclusion of other vertical forces, only the gravitational force was included.

**Model calculations**

First, the receptors were distributed over the surface of sphere. The initial separation distance for a sphere from the wall was randomly selected from the Boltzmann probability distribution for occupying a state in a gravitational field using a Monte Carlo approach:

$p\left(\delta\right)=Ce^{-\frac{E\left(\delta\right)}{k_{B}T}}$. (S1.1)

Where C is a normalization constant, and the potential energy, E, is given by:

$E\left(\delta\right)=\int_{\delta_{\min}}^{\delta} (\rho_{s}-\rho_{w})\frac{4}{3}\pi R^{3}gdz$. (S1.2)

The symbols and parameter values are given in Table 1. Next, a time step size was selected. Since capturing accurate motions of the microbead relative to the molecular bond length would be important for accurate bond force calculations, and a characteristic maximum velocity for the sphere would be the wall shear rate times the radius, the time step size was set according to:

$\Delta t=\frac{0.01\lambda}{RS}$. (S1.3)

The calculations for each time step were then performed in the following order:

1. The forces and torques acting on the sphere due to gravity, existing bonds, and fluid flow were added together.
2. Dimensionless damping factors were calculated. These are correction factors to the Stokes solutions for rotational and linear drag on the particle moving in an incompressible, viscous fluid due to the presence of the wall. More detail on their implementation is given in the next section.
3. A diffusive component to the in-plane (nonvertical) linear motions and rotations was calculated. More detail can be found in the next section.
4. The expected linear and angular velocities were then calculated with the mobility matrix:

$\vec{v}=\underline{M}\vec{f}$. (S1.4)

The calculation is described in greater detail in the next section.

1. The vertical displacement of the sphere was then separately calculated. This was necessary because, unlike the damping factor for lateral motions, the damping factor in the vertical direction is a function of its own coordinate. We used a method similar to that used previously [5], except we allow for the bond forces in the calculation and a variable vertical step size. Greater detail on the calculation is given in the next section.
2. The number concentration of free ligand in the contact patch, the area within a maximally extended bond length of the sphere, was calculated based on the assumed total ligand site density and the number of receptors bound to ligand on the sphere. Since the ligand was assumed to be immobilized to the surface, the anchorage point could not diffuse. The site density of ligand in the contact patch could then be calculated from:

$n_{L}=n_{L}^{O}-\frac{N_{RL}}{\left(\lambda-\delta\right)2\pi R}$. (S1.5)

The binding rate for each receptor on the surface of the sphere to the ligand-coated surface was calculated from (1,2). For molecular area confinement, k_f_^T^, was normalized based on k_f_ so both (1,2) would yield the same average reaction rate across the contact patch for a sphere touching the wall to within surface roughness limitations:

$k_{f}=\frac{1}{\left(\lambda-\delta_{\min}\right)2\pi R}\int_{0}^{2\pi} \int_{r\left(h=\delta_{\min}\right)}^{r\left(h=\lambda\right)} k_{f}^{T}\frac{\left(\lambda^{2}-h^{2}\left(r\right)\right)}{\lambda^{2}}rdrd\theta$. (S1.6)

Each unbound receptor on the sphere was tested for bond formation assuming a Poisson arrival process. The cumulative distribution function of a single receptor forming a bond during a time step then became:

$P\left(t\leq\Delta t\right)=1-e^{-k_{on}\Delta t}$. (S1.7)

This probability was tested by Monte Carlo simulation. If bond formation was successful, a point directly below the receptor in the plane of the ligand-coated surface was assigned as the ligand anchorage point of the nascent receptor-ligand complex for the next time step. Note that no bonds formed under tension in the model.

1. The dissociation rate for existing receptor-ligand complexes was computed using (3,4) and they were tested for dissociation, using Monte Carlo simulation and a rate expression similar to (S1.7).
2. The sphere was then moved according to the linear and angular velocities calculated in steps 4 and 5. Gimbal lock problems were avoided by using quaternions to track the sphere’s rotations [9].
3. The bond status of each receptor was updated based on the results of steps 6 and 7.
4. The length of existing bonds was calculated according to the updated Cartesian coordinate of the receptor on the sphere and the Cartesian coordinate of the ligand on the surface. The force on each bond was calculated from (5,6). The resulting torque on the sphere at each receptor by each bond was calculated:

. (S1.8)

The time step was then incremented and calculations repeated from step 1. Tracking and testing unbound receptors for formation carried a heavy computational cost, but was tractable. A simulation of 10 s of binding interactions for a 10 µm-diameter sphere with 50 receptors per µm^2^ typically took 30 hours on one single-core processor of a node of the Dogwood Linux Cluster at the University of Virginia. Each node was equipped with two 3GHz Intel Xeon EMT CPUs and 3GB of RAM. Simulation time roughly scaled with the number of receptors on the sphere.

**Microsphere convective and diffusive motion**

The solutions to the problems of the forces and torques exerted on a sphere moving or rotating at a prescribed velocity in an incompressible, Newtonian fluid can be found in textbooks on fluid dynamics [10]. The presence of an infinite surface, or wall, near the sphere alters the solution and creates directionality: the resistance to motion parallel to the surface is different from motion away. It also couples rotation about an axis parallel to the wall with an imparted translational force parallel to the wall. The problems of the different motions have been developed separately. Force and torque balances in each direction take the form:

$f_{i,applied}+f_{t}^{S}f_{it}^{*}\left(\frac{\delta}{R}\right)v_{i}+f_{t}^{S}Rf_{ir}^{*}\left(\frac{\delta}{R}\right)\omega_{i}=0$, (S1.9)

$t_{i,applied}+\frac{t_{r}^{S}}{R}t_{it}^{*}\left(\frac{\delta}{R}\right)v_{i}+t_{r}^{S}t_{it}^{*}\left(\frac{\delta}{R}\right)\omega_{i}=0$. (S1.10)

The superscript S denotes the corresponding Stokes solution without a wall and the superscript asterisk indicates the dimensionless “correction” factor that must be applied due to the wall, taken to be positive. For example, to move a sphere in the x-direction with a velocity, v_x_, and with a right-handed angular velocity about the perpendicular axis, ω_y_, requires an applied force:

$f_{x}=6\pi\mu R\left|f_{x,t}^{*}\left(\frac{\delta}{R}\right)\right|v_{x}-6\pi\mu R^{2}\left|f_{x,r}^{*}\left(\frac{\delta}{R}\right)\right|\omega_{y}$. (S1.11)

The absolute value was taken to clarify directionality. The dimensionless coefficients can be calculated with different relations. We detail our calculation method for each below.

- f_xt_^*^, f_yt_^*^; t_xt_^*^, t_yt_^*^; f_xr_^*^, f_yr_^*^; t_xr_^*^, t_yr_^*^: These quantities were presented previously [11,12]. A numerical tabulation of the values over intermediate gap ranges is given, and we have opted to use cubic spline interpolation of the table values since this method has been used, with some experimental validation, to calculate particle trajectories for related problems [13]. We used the M3A method of cubic Hermite spline interpolation since it yields third-order accurate, monotonic splines [14]. Goldman et al. also give expressions for the asymptotic solutions for the near (small δ:R) and far-wall (large δ:R) conditions, which were employed in the limit but have been omitted here for brevity.
- t_zr_^*^: The solution was derived by Jeffery [15]. The analytical expression employed was:

$t_{zr}^{*}=-\sum_{m=1}^{\infty} \csc h^{3}\left(m\ln\left(\frac{\delta+R}{R}+\sqrt{\left(\frac{\delta+R}{R}\right)^{2}-1}\right)\right)$. (S1.12)

- f_zt_^*^: This quantity was originally derived by Brenner [16], although the series expression was slow to converge computationally. Excellent approximations to this solution are available. For very small gaps we used the expression as employed in lubrication theory [17]:

$f_{zt}^{*}=-\frac{\left(1-\frac{\frac{\delta}{R}}{\frac{\delta}{R}+1}\right)^{2}}{\frac{\delta}{R}}$. (S1.13)

Pierres et al. [5] give an expression that fits Brenner’s result very well for most of the gap sizes encountered in the simulation runs:

$f_{zt}^{*}=-e^{0.0057685\ln^{3}\left(\frac{\delta}{R}\right)+0.092235\ln^{2}\left(\frac{\delta}{R}\right)-0.52669\ln\left(\frac{\delta}{R}\right)+0.76952}$. (S1.14)

We switch between the expressions at gap to radius ratio of 9.389736 × 10^-4^, where the two equations are equal.

Once the dimensionless damping factors were calculated, the angular and linear velocities in a time step were calculated with the mobility matrix. The force required to move an object with a specified velocity is proportional to the hydrodynamic drag. For clarity, the expression employed was:

$\left[\begin{matrix} v_{x} \\ v_{y} \\ v_{z} \\ \omega_{x} \\ \omega_{y} \\ \omega_{z} \end{matrix}\right]=-1*\left[\begin{matrix} -6\pi\mu R\left|f_{xt}^{*}\left(\frac{\delta}{R}\right)\right| & 0 & 0 & 0 & 6\pi\mu R^{2}\left|f_{xr}^{*}\left(\frac{\delta}{R}\right)\right| & 0 \\ 0 & -6\pi\mu R\left|f_{yt}^{*}\left(\frac{\delta}{R}\right)\right| & 0 & -6\pi\mu R^{2}\left|f_{yr}^{*}\left(\frac{\delta}{R}\right)\right| & 0 & 0 \\ 0 & 0 & -6\pi\mu R\left|f_{zt}^{*}\left(\frac{\delta}{R}\right)\right| & 0 & 0 & 0 \\ 0 & -8\pi\mu R^{2}\left|t_{xt}^{*}\left(\frac{\delta}{R}\right)\right| & 0 & -8\pi\mu R^{3}\left|t_{xr}^{*}\left(\frac{\delta}{R}\right)\right| & 0 & 0 \\ 8\pi\mu R^{2}\left|t_{yt}^{*}\left(\frac{\delta}{R}\right)\right| & 0 & 0 & 0 & -8\pi\mu R^{3}\left|t_{yr}^{*}\left(\frac{\delta}{R}\right)\right| & 0 \\ 0 & 0 & 0 & 0 & 0 & -8\pi\mu R^{3}\left|t_{zr}^{*}\left(\frac{\delta}{R}\right)\right| \end{matrix}\right]^{-1}\left[\begin{matrix} f_{x} \\ f_{y} \\ f_{z} \\ t_{x} \\ t_{y} \\ t_{z} \end{matrix}\right]$. (S1.15)

Observe the magnitudes of the damping factors were taken and the appropriate sign added for clarity. A positive sign is required in front of the t_yt_^*^ and f_xr_^*^ term due to the assumed positive nature of right-handed rotation. Fluid shear and bond forces contributed to f_x_, bond forces contributed to f_y_, colloidal and bond forces contributed to f_z_, bond torque contributed to t_x_, fluid shear and bond torque contributed to t_y_, and bond torque contributed to t_z_.

The translation and rotation could then be calculated as v_i_Δt and ω_i_Δt, but this would neglect the effects of diffusion. The partial differential equation governing the probability distribution function for diffusive motion is [18]:

$\frac{\partial f}{\partial t}=k_{b}Tm\frac{\partial^{2}f}{\partial\eta^{2}}$. (S1.16)

Here, m is the mobility of the particle, which would be (6πμR)^-1^ for the familiar case where η refers to a coordinate of lateral position and the resistance to motion is that of a sphere in a viscous fluid (excluding consideration of the wall). If one only considers motions where m is not a function of η and the position at initial time point is fixed at zero, then the partial differential equation is solved to yield:

$f\left(\eta,t\right)=\frac{1}{\sqrt{4t\pi k_{b}Tm}}e^{-\frac{\eta^{2}}{4tk_{b}Tm}}$. (S1.17)

If one fixes the time interval as Δt, then the probability distribution function for η at the end of the time step Δt is a zero-mean Gaussian distribution:

$f\left(\eta\right)=\frac{1}{\sigma_{\eta}\sqrt{2\pi}}e^{-\frac{\eta^{2}}{2\sigma_{\eta}^{2}}}$. (S1.18)

The standard deviation is given by:

$\sigma_{\eta}=\sqrt{2\Delta tk_{B}Tm}$. (S1.19)

For example, the standard deviation for diffusive motion in the x-direction would be:

. (S1.20)

This form of the solution suggests a method for adding diffusive effects into the simulation. Calculate the standard deviation of the diffusive component of the motion from (S1.20) and use a normally-distributed random number generator to select the diffusive component to the change for the current time step. Then, add the result to new positions calculated based on the “deterministic” force balance with the aid of (S1.15). However, a lateral displacement due to thermal motion in the absence of a counteracting torque should result in rotation due to the wall; likewise, a rotational motion due to thermal rotation in the absence of a stabilizing force should result in lateral motion due to the wall. The relation between motions predicted by (S1.15) should hold for the diffusive component of the motion as well. To ensure the bead motion distributions obey the motion coupling effect of the wall with lateral and rotational diffusion, we calculated a coupling factor necessary to displace the bead an equivalent amount to thermal motion based on the relation:

$\eta=m\zeta\Delta t$. (S1.21)

Here, ζ has dimensions of force or torque and η was again either a translational or angular displacement. The coupling factor, ζ, is then also normally distributed with standard deviation:

$\sigma_{\zeta}=\sqrt{\frac{2k_{B}T}{m\Delta t}}$. (S1.22)

The results suggest an alternative method of solution. Calculate a coupling factor according to (S1.22) with a normally-distributed random number generator, add the resulting force to the external forces acting on the sphere in (S1.15), and then calculate the motion of the sphere. Note that due to the nature of Brownian motion the velocity would not be constant over a time step. Given the randomly calculated end coordinates due to thermal component of motion during a time step, it is worth noting there are a variety of paths the sphere might take to get there. The method holds to be analytically correct despite a variety of paths the particle might take between the start and end points.

Because the vertical mobility is a function of its own coordinate, the vertical translation must be treated in a different manner. This should be apparent when attempting to derive (S1.17) from (S1.16). A solution method for this problem has been previously developed [5], and we implement a similar approach with some small modification. The sphere can move up or down due to z-directed external forces or diffusion in a time step. The z-levels to test during each time step were discretized. Characteristic frequencies for directional vertical steps due to diffusion or applied forces can be respectively calculated from:

$\nu=\frac{k_{B}T}{6\pi\mu R\left|f_{z}^{*}(\frac{\delta}{R})\right|\left(\Delta h\right)^{2}}$, (S1.23)

$\nu=\frac{\left|F_{z}\right|}{6\pi\mu R\left|f_{z}^{*}(\frac{\delta}{R})\right|\Delta h}$. (S1.24)

The z-level discretization would only be problematic only if the magnitude of z-level spacing, Δh, was small enough to limit the sphere’s vertical motion artificially by the simulation. We allowed the vertical step size to vary keeping this requirement in mind. The requirement was:

$\frac{\Delta h}{\Delta t}\leq\nu$. (S1.25)

The solution to the resulting quadratic equation for a level spacing that results changes with 1% of the characteristic frequency was:

$\Delta h\left(up|down\right)=\left(\frac{\left|F_{z}\left(up|down\right)\right|+\sqrt{\left|F_{z}\left(up|down\right)\right|^{2}+\frac{0.01\pi\mu Rk_{B}T}{\Delta t}}}{2*0.01*6\pi\mu R\left|f_{z}^{*}\left(\frac{\delta}{R}\right)\right|}\right)\Delta t$. (S1.26)

This level spacing was calculated separately for the up and down directions, evaluating the vertical damping factor at the current height. For clarity, Δh(up) did not equal Δh(down). In the expressions, F_z_ was assumed zero in the direction for which the summed vertical force was not acting and equal to its magnitude in the direction of action. The quantity, F_z_, was necessarily nonzero for the Δh(down) calculation due to gravity. If the predicted level spacing in the down direction took the sphere closer to the wall than the assumed minimum due to surface roughness, the downward level spacing was set equal to the remaining distance to the minimum height. When the sphere was at the height set by surface roughness, successful steps to lower coordinates resulted in the sphere resuming the minimum height.

Three discrete vertical levels were created based the optimal spacings, one in the upwards direction, one at the same level, and one in the down direction. Unlike the method of Pierres et al. [5], all factors contributing to upward motion were lumped together as one frequency and all factors contributing to downward motion were lumped together as a second frequency. For each time step, the motion was tested for a step up followed by a test for a step down. For example, the frequency of a step up was calculated according to:

$\nu\left(up\right)=\frac{k_{B}T}{6\pi\mu R\left|f_{z}^{*}\left(\frac{\delta+\frac{\Delta h\left(up\right)}{2}}{R}\right)\right|\Delta h\left(up\right)^{2}}+\frac{\left|F_{z}\left(up\right)\right|}{6\pi\mu R\left|f_{z}^{*}\left(\frac{\delta+\frac{\Delta h\left(up\right)}{2}}{R}\right)\right|\Delta h\left(up\right)}$. (S1.27)

Of course, F_z_(up) was necessarily zero since steric and electrostatic repulsion were neglected, but the term is shown here for clarity since it was important for F_z_(down) and to facilitate future extension to cases where an upward-directed force might be incorporated into the model. The probability for stepping up in the first sub-step could then be calculated according to:

$P\left(up\right)=1-e^{-\nu\left(up\right)\Delta t}$. (S1.28)

Next, the probability of stepping down was calculated, either back to the initial level or to the lower level depending on the result of the previous sub-step. The probabilities of ending up at the higher, same, or lower level at the end of the step were then calculated assuming the subsequent steps occurred independently, and Monte Carlo was used to establish the new vertical position of the bead. The accuracy of this method for modeling the effects of forces and diffusion was tested by demonstrating it recreates the Boltzmann distribution at sufficiently long time scales, see Figure 3.

**Pause time analysis of simulation results**

To compute the apparent k_off_, a method similar to that used for experimental analysis was employed. A threshold velocity value was selected as a cutoff. The pause duration was considered to be the length of time the velocity remained below the selected value. Based on previously published observations for noise in a velocity signal [19], a reasonable event cutoff was 6.5 µm/s assuming experiments were performed at 40× and 250 fps. The pause time distribution from which the apparent k_off_ is measured is generally modeled as a constant rate Poisson arrival process, and the cumulative density function can be modeled as an exponential approach function. Several methods of Poisson parameter estimation exist, the simplest of which would be to measure the expected value. It was opted to analyze logarithms of the one minus the pause duration percentile, which would be a linear transform for exponentially distributed cumulative probability data, in better analogy with experiment. Three simulations were run for 10 s of simulated time with each of the biomolecular assumptions. Simulation results were sampled at 250 fps and the data were pooled.

**Skip distance analysis of simulation results**

To better test the validity of the multi-component Poisson model, the likelihood ratio test for mixed Poisson processes was employed [20]. Regression estimates were used as the maximum likelihood estimators. Qualitatively the data were fit much better by two-component processes than single-component processes in all of the cases considered, but this result was only statistically significant at the 95% confidence level with 1× to 2× relative ligand densities and higher. However, the assumption of a two-component model was not likely inappropriate at low ligand densities. Instead, the result was likely due to the smaller number of pause events observed at dilute ligand concentrations. Indeed, Karlis and Xekalaki [20] found sample sizes of 500 events were sometimes needed to ensure the accurate deduction of a two-component model over the incorrect deduction of a one-component model. The two-component analysis results were therefore compiled for Table S1 at low ligand site densities. Interestingly, at the highest ligand site densities, a three-component model was statistically justified. The shortest two of the three components were within the length scale of a contact patch. The result suggests statistical evidence of relative receptor spacing might be estimated from an equivalent experimental analysis at high ligand densities.

**Diffusive motion of a tethered sphere**

The size of the apparent velocity fluctuations in Figures 4A,B are roughly ± 5 µm/s and ± 15 µm/s, respectively. When experimentally tracking micron-scale objects using high spatiotemporal-resolution videomicroscopy, the noise of the video acquisition usually limits accuracy of frame-to-frame changes in the position of the object’s center to within roughly ± 0.14 pixels [19,21]. Given a camera pixel element size of 7.4 µm, an optical magnification of 200× would be needed to reliably detect differences in the fluctuations above noise, at least with the detection system previously characterized [19]. The standard deviation of the sampled velocity due to diffusive fluctuations should scale inversely with the square root of time (divide (S1.20) by the frame interval). It has been noted that for a video acquisition system the noise in the frame-to-frame positional change is reduced with increased optical magnification [19]. Assuming the positional noise reduction is linear with magnification, the result suggests it would be theoretically possible to detect differences in biomolecular stiffness from velocity fluctuations at 60 fps with 60× magnification.

Determining differences in tether stiffness by differences in the V_S,X_ fluctuations during a tether event, as shown in Figure 4, could be challenging. Such a measurement would have the best chance of success if the surface was molecularly smooth, such as with mica, or the interaction was shielded with an ideal blocker. It is also important to note that the difference in the assumed bond stiffness in Figures 4A,B was large with a relatively small resulting change in V_S,X_. Two additional characteristics might be used to achieve confidence in the velocity signal when attempting to make the measurement: the asymmetric nature of the restricted diffusion shown in Figure 4A, as well as a homogenous distribution of the temporal Fourier frequency transform [19]. The noise present in the tracking system will have to be carefully characterized if such a measurement is attempted.

**Comparison of simulation to tracking results**

Video was tracked using a centroid tracker with object segmentation, **MCShape**, similar to the previously validated **CMorph** algorithm [19]. The method implemented was similar to that previously reported, except the phase contrast acquisition method used by Dr. Park was exploited to segment the object from the image intensity rather than from the gradient. The MATLAB tracking function is available at the laboratory web site, <http://bme.virginia.edu/lawrence/>.

The high-frequency wobbles in Figures 7B,C apparent with the object-segmentation centroid-tracking method are not apparent with the sum-of-absolute-difference method in Figure 7A or in the simulation results in Figure 7D. The fluctuations likely do not correspond to physical wobble due to bond formation because they do not correspond to time points when the molecular tethers are stressed, with drops in V_S,X_, as can be seen in Figure 7D. It appears the object-segmentation centroid tracking method has a significant amount of noise which impacts the appearance of the velocity waveform. On the other hand, the results with the sum-of-absolute-difference tracking method exhibit less wobble than predicted by the simulation. There are three possible reasons for the discrepancy. Because the sum-of-absolute-difference tracking method requires an interpolation algorithm to achieve sub-pixel accuracy, it is possible there is some quantization effect preventing the algorithm from detecting small shifts in the perpendicular centroid location. Positional bias might cause underestimation of wobble motion. In Figure 7B, it appears the centroid tracking method picks up wobble similar to the simulation that is not as apparent in the sum-of-absolute-differences results. A second possibility is the simulation may overpredict wobble, which would suggest bond formation events are more strongly impacted confinement enhancement than assumed in (1,2). A third possibility is some physical nonideality, like friction, prevents the sphere from sliding into the optimal mechanical configuration. A thorough theoretical investigation of motions detectable with the experimental system would help discriminate between these possibilities. Two-dimensional artificial images created from continuous splines could be created from the simulation results. The artificial images could be discretized with a camera pixel sampling grid, a realistic amount of camera noise could be added in, and the artificial rolling movies could be tracked to help decide between these possibilities. A similar investigation of tracking particles has been done previously, although the particle motions were specified rather than simulation outputs and the artificial images assumed the appearance of fluorescent particles [21]. It is important to point out that movies were taken with a 20× objective and went through an intermediate analog conversion. It should be possible to get much better quality data with a modern camera system allowing for all digital acquisition and using higher optical magnification. Additionally, studies exploiting the measurement of wobble motion to deduce molecular parameters might employ larger beads to enhance the lateral motion.

It was noted that although the simulation matched particle slowing well in Figure 7, it did not match acceleration well. The experiment exhibited an acceleration lag the model did not capture. The source of the lag could not be due to lifting of the particle away from the wall. The roughness was set to a relatively small value in the simulation and the steady simulated velocity matched the steady experimental velocity well with this very near-wall case. Furthermore, the lag was not likely due to any inertial component that has been neglected in the simulation. The time scale where inertia would become important was estimated:

$\tau=\frac{L^{2}\rho_{w}}{\mu}$. (S1.29)

Here, τ is the inertial time scale, L is the relevant length dimension, ρ_w_ is the fluid density, and μ is the viscosity. The sphere’s size should be the relevant length scale since the fluid must rotate with the sphere’s surface due to the no-slip condition. The radius was much larger than the gap and was a conservative length scale to consider. The relevant time scale was still only on the order of microseconds, much smaller than the millisecond sampling rate. The lag in acceleration observed in the experiment was therefore most likely due to some non-ideal interaction with the surface, perhaps the microbead or an adsorbed protein layer was sliding in intimate contact with the ligand surface. A similar finite time period required for particle motion was observed with detachment from ligand-presenting accumulation strips [3]. It is also possible the lag was due to short-lived bonds, but the regularity of the motion and scaling of distance with wall shear noted previously [3] suggested this was not the case.

**References**

1. King MR, Hammer DA (2001) Multiparticle adhesive dynamics: hydrodynamic recruitment of rolling leukocytes. Proc Natl Acad Sci USA 98: 14919-14924.

2. Bhatia SK, King MR, Hammer DA (2003) The state diagram for cell adhesion mediated by two receptors. Biophys J 84: 2671-2690.

3. Schmidt BJ, Huang P, Breuer KS, Lawrence MB (2008) Catch Strip Assay for the Relative Assessment of Two-Dimensional Protein Association Kinetics. Anal Chem 80: 944-950.

4. Huang P, Guasto JS, Breuer KS (2006) Direct measurement of slip velocities using three-dimensional total internal reflection velocimetry. J Fluid Mech 566: 447-464.

5. Pierres A, Benoliel AM, Zhu C, Bongrand P (2001) Diffusion of microspheres in shear flow near a wall: use to measure binding rates between attached molecules. Biophys J 81: 25-42.

6. Bell GI, Dembo M, Bongrand P (1984) Cell adhesion. Competition between nonspecific repulsion and specific bonding. Biophys J 45: 1051-1064.

7. Israelachvili JN (1985) Intermolecular and Surface Forces with Applications to Colloidal and Biological Systems. London: Academic Press.

8. Bongrand P, Bell GI (1984) Cell-Cell Adhesion: Parameters and Possible Mechanisms. In: Perelson AS, DeLisi C, Wiegel FW, editors. Cell Surface Dynamics: Concepts and Models. New York: Marcel Dekker, Inc. pp. 459-493.

9. Horn BKP (1987) Closed-Form Solution of Absolute Orientation Using Unit Quaternions. J Opt Soc Am A 4: 629-642.

10. Deen WM (1998) Analysis of Transport Phenomenon. New York: Oxford University Press.

11. Goldman AJ, Cox RG, Brenner H (1967) Slow viscous motion of a sphere parallel to a plane wall I: Motion through a quiescent fluid. Chem Eng Sci 22: 637-651.

12. Goldman AJ, Cox RG, Brenner H (1967) Slow viscous motion of a sphere parallel to a plane wall II: Couette flow. Chem Eng Sci 22: 653-660.

13. Schmidt BJ, Sousa I, van Beek AA, Bohmer MR (2008) Adhesion and ultrasound-induced delivery from monodisperse microbubbles in a parallel plate flow cell. J Control Release 131: 19-26.

14. Huynh HT (1993) Accurate Monotone Cubic Interpolation. SIAM J Numer Anal 30: 57-100.

15. Jeffrey GB (1915) On the Steady Rotation of a Solid of Revolution in a Viscous Fluid. Proceedings of the London Mathematical Society 14: 327-338.

16. Brenner H (1961) The slow motion of a sphere through through a viscous fluid towards a plane surface. Chem Eng Sci 16: 242-251.

17. Georges JM, Millot S, Loubet JL, Tonck A (1993) Drainage of Thin Liquid-Films between Relatively Smooth Surfaces. Journal of Chemical Physics 98: 7345-7360.

18. Einstein A (1956) Investigations on the Theory of Brownian Movement: Dover Publications, Inc.

19. Schmidt BJ, Paschall CD, Guilford WH, Lawrence MB (2007) High Resolution Optical Tracking to Identify Adhesive Events in Vitro; Pacific Grove, CA. ACSSC: 1856-1860.

20. Karlis D, Xekalaki E (1999) On testing for the number of components in a mixed Poisson model. Ann I Stat Math 51: 149-162.

21. Cheezum MK, Walker WF, Guilford WH (2001) Quantitative comparison of algorithms for tracking single fluorescent particles. Biophys J 81: 2378-2388.
