# Supplementary material for: Nano-motion Dynamics are Determined by Surface-Tethered Selectin Mechanokinetics and Bond Formation
Source: PLoS Comput Biol. 2009 Dec 18;5(12):e1000612. doi: 10.1371/journal.pcbi.1000612 (PMC2787012; doi:10.1371/journal.pcbi.1000612)
Supplement: Protocol S2 — Additional lateral transport considerations. The negligible effect of lateral transport on the bond formation rate under the simulated experimental conditions is discussed further. An alternative model of encounter complex and bond formation that could be used to incorporate lateral transport effects is presented. (0.03 MB DOC) [file pcbi.1000612.s002.doc]

**Protocol S2. Additional lateral transport considerations.**

In the model formulation presented, receptors were treated as discrete elements firmly tethered to the sphere’s surface, and ligands were considered to be firmly tethered to the target surface. For a sphere touching the wall, there would be an effective search radius of 92 nm between the receptor anchor point and ligand anchor point. One receptor-ligand complex could occupy up to 0.03 µm^2^. An encounter complex has been described previously as a receptor and ligand that have approached closely enough to bind but have not yet done so [1,2]. For the aid of discussion, we define the tether-point encounter complex. A receptor and ligand, each confined by a tether point to a separate surface, approach such that their anchor point to their respective surface in three-dimensional space are within an unstrained molecular bond length of each other. Because the simulated baseline ligand density was approximately 100 molecules/µm^2^, a given receptor should be within a suitable distance of three ligands to form a bond. The receptor should be involved in three tether-point encounter complexes. To a first approximation, macromolecular transport parallel to the ligand surface should not be rate limiting. The wait for the lateral arrival of at least one ligand’s tether point to approach within 92 nm of the receptor’s tether point should not be significant. This does not presuppose vertical transport would not be important. Vertical transport was functionally included in the on-rate through (1,2).

A rate limiting step for formation could either be local motion of the receptor and ligand binding pockets on their molecular stalks to find each other, or finer re-orientation of the molecular head groups once they happen upon a suitable encounter. To aid discussion, the binding-pocket encounter complex is defined as a pair of receptor and ligand reactive head groups that are close enough to bind but have not yet made the finer orientation and molecular subdomain adjustments to finish binding. If the transition from tether-point to binding-pocket encounter complex was an important step in determining the rate (Figure S6), it is possible the apparent binding rate could be enhanced if the receptor had, for example, six ligand anchorage points within 92 nm rather than three. The model does capture this behavior by inclusion of the linear dependence of bond formation rates for a receptor on free ligand density.

The effects of sphere motion on macromolecular transport with a surface-immobilized ligand might be most easily formulated by dealing with the problem in a stochastic framework. This could be implemented in the model by adding an additional variable for each receptor to track the number of tether-point encounter complexes the receptor would be engaged in. During each time step, a receptor could form additional tether-point encounter complexes with the rate:

$k_{TPEC}^{+1}=H\left(\lambda-z_{R}\right)\left(\int_{-\pi/2}^{\pi/2} V_{XYslip,R}\sqrt{\lambda^{2}-z_{R}^{2}}\cos\left(\theta\right)d\theta-2\pi z_{R}v_{z,R}H\left(-v_{z,R}\right)\right)n_{L}$. (S2.1)

Here, V_XYslip,R_ is the X-Y planar component of the slip velocity of the receptor on the sphere’s surface, which can easily be calculated based on the sphere’s rotational and lateral motion and the coordinates of the receptor on the sphere’s surface. Also, v_Z,R_ is the z-component of the velocity for receptor relative to the surface. Motion would also carry potential binding partners out of the tether point-encounter complexes. The reverse rate would be:

$k_{TPEC}^{-1}=H\left(\lambda-z_{R}\right)\left(\frac{\pi V_{XYslip,R}}{\int_{-\pi/2}^{\pi/2} 4\sqrt{\lambda^{2}-z_{R}^{2}}\cos^{3}\left(\theta\right)d\theta}+2\pi z_{R}v_{z,R}H\left(v_{z,R}\right)\right)N_{NTPEC,R}$. (S2.2)

Here, N_TPEC,R_ is the current number of tether point encounter complexes for the receptor under consideration. With this approach, each receptor would then have a bond formation rate that scaled with N_TPEC,R_, which would effectively lump together the rates of binding-pocket encounter complex formation and intrinsic bond formation.

For the purposes of illustration, assume the unbound ligand concentration is fixed. Also assume the z-coordinate of a receptor on the sphere is fixed at the ligand surface but can slide over the substrate at constant velocity. Then it is possible to derive:

$N_{TPEC,R}\left(t\right)=\left(N_{TPEC,R}\left(t=0\right)-\frac{32}{3}\lambda^{2}n_{L}\right)e^{-\frac{3}{16}\frac{V_{XYslip,R}}{\lambda}t}+\frac{32}{3}\lambda^{2}n_{L}$. (S2.3)

This result illustrates that the lateral slip velocity does not influence the average number of tether-point encounter complexes a receptor engages in for a molecular system where the anchor points are immobilized. Rather, the lateral slip velocity would influence how quickly the system re-equilibrates to changes. The average number of tether-point encounter complexes is determined by the molecular bond length, ligand density, and the vertical coordinate.

An analysis of the time until bond formation for a particle undergoing thermal fluctuations in linear shear was performed by Korn and Schwarz [3]. Rather than considering reaction, the focus was to use reaction geometry to characterize the mean first passage time until there was a molecular encounter between patches of receptor on the sphere and ligand on the surface. Consider that in the present analysis, the 6 µm-diameter and 10 µm-diameter spheres were in contact with the surface 67% and 99% of the time, respectively, and there was no initial sedimentation. The spheres were released at the initial time according to their equilibrium distribution. In terms of the parameters used by Korn and Schwarz, the conditions in modeled through the latter portion of the present investigation were: an x-direction Péclet number (Pe) of 26,090, a normalized receptor density (ρ_r_) of 2.6, and 30,000 receptors on the sphere (N_r_). Essentially, at least one receptor was always within a suitable distance to form an encounter complex for the simulations with 10 µm-diameter spheres employed through much of the present analysis. The implication is that the transition to a binding-pocket encounter complex or to complete binding is critical. Improved surfaces, receptor immobilization methods, and molecular engineering of the receptors will help to unravel fundamental questions related to encounter complex dynamics, especially when coupled with the quantitative analysis methodology developed here.

**References**

1. Shoup D, Szabo A (1982) Role of diffusion in ligand binding to macromolecules and cell-bound receptors. Biophys J 40: 33-39.

2. Lauffenburger DA, Linderman JJ (1993) Receptors: models for binding, trafficking, and signaling. New York: Oxford University Press. 365 p.

3. Korn CB, Schwarz US (2007) Mean first passage times for bond formation for a Brownian particle in linear shear flow above a wall. J Chem Phys 126: 17.
