# Supplementary material for: Nano-motion Dynamics are Determined by Surface-Tethered Selectin Mechanokinetics and Bond Formation
Source: PLoS Comput Biol. 2009 Dec 18;5(12):e1000612. doi: 10.1371/journal.pcbi.1000612 (PMC2787012; doi:10.1371/journal.pcbi.1000612)
Supplement: Table S1 — Compilation of simulation results. (0.31 MB DOC) [file pcbi.1000612.s003.doc]

Table S1. Compilation of simulation results.

| Dissociation Model Input | Cluster Valence | Statistical Point Estimate k_off_ | Cnf Model | Receptor Cluster Density  × 95 | Ligand Cluster Density × 90 | Pause Statistics | | | Skip Statistics | | | | | | | | | | | | | | | | | Velocity | |
| --- | --- | --- | --- | --- | --- | --- | --- | --- | --- | --- | --- | --- | --- | --- | --- | --- | --- | --- | --- | --- | --- | --- | --- | --- | --- | --- | --- |
|  |  |  |  |  |  | Apparent k_off_ | s_koff_ | R^2^ | Optimal Mixed Poisson Process | Short Skip Fraction | 95% CI | | Short Skip Distance | 95% CI | | Long Skip Distance | 95% CI | | Medium Skip Fraction | 95% CI | | Medium Skip Distance | 95% CI | | R^2^ | Average | Standard Deviation |
|  |  | s^-1^ |  | sites/µm^2^ | sites/µm^2^ | s^-1^ | s^-1^ |  |  |  |  |  | µm | µm | | µm | µm | |  |  |  | µm | µm | |  | µm/s | µm/s |
| Bell slip Park et al. [1] | 1 | 12.6 | CP | 1× | 1× | 12.0 | 0.06 | 0.995 | 2 | 0.62 | 0.61 | 0.64 | 0.69 | 0.67 | 0.74 | 12.89 | 12.20 | 13.59 |  |  |  |  |  |  | 0.998 | 38.3 | 5.54 |
|  |  |  | MA | 1× | 1× | 11.6 | 0.14 | 0.976 | 2 | 0.71 | 0.70 | 0.72 | 0.58 | 0.56 | 0.60 | 15.23 | 13.95 | 16.50 |  |  |  |  |  |  | 0.996 | 32.3 | 2.62 |
| Bell slip  Edmondson et al. [2] | 2 | 20.1 | CP | 1× | 1× | 12.6 | 0.09 | 0.985 | 3^a^ | 0.45 | 0.40 | 0.50 | 0.20 | 0.19 | 0.22 | 14.48 | 12.86 | 16.09 | 0.33 | 0.29 | 0.37 | 0.98 | 0.79 | 1.16 | 0.998 | 30.1 | 3.70 |
|  |  |  | MA | 1× | 1× | 12.1 | 0.09 | 0.985 | 3 | 0.36 | 0.32 | 0.41 | 0.14 | 0.12 | 0.15 | 14.62 | 12.26 | 16.98 | 0.45 | 0.41 | 0.49 | 0.70 | 0.61 | 0.80 | 0.997 | 28.2 | 18.97 |
| Catch-slip  Marshall et al. [3,4] | 1 | 100 | CP | 1× | 1/2× | 51.7 | 0.70 | 0.987 | 2^b^ | 0.26 | 0.25 | 0.28 | 1.81 | 1.59 | 2.03 | 46.17 | 44.60 | 47.73 |  |  |  |  |  |  | 0.997 | 98.0 | 0.45 |
|  |  |  | MA | 1× | 1/2× | 66.1 | 1.41 | 0.972 | 2^c^ | 0.19 | 0.16 | 0.21 | 3.24 | 2.15 | 4.34 | 43.84 | 41.53 | 46.15 |  |  |  |  |  |  | 0.994 | 100.6 | 2.90 |
|  | 1 | 100 | CP | 1× | 2/3× | 64.6 | 1.31 | 0.965 | 2^d^ | 0.22 | 0.20 | 0.24 | 3.24 | 2.72 | 3.76 | 34.71 | 33.49 | 35.99 |  |  |  |  |  |  | 0.997 | 95.9 | 0.47 |
|  |  |  | MA | 1× | 2/3× | 70.3 | 1.73 | 0.957 | 2^e^ | 0.05 | 0.03 | 0.07 | 0.30 | -0.56 | 1.16 | 33.15 | 31.82 | 34.47 |  |  |  |  |  |  | 0.987 | 97.9 | 0.20 |
|  | 1 | 100 | CP | 1× | 1× | 55.5 | 0.94 | 0.970 | 2^f^ | 0.07 | 0.06 | 0.08 | 0.40 | 0.23 | 0.57 | 21.45 | 21.10 | 21.80 |  |  |  |  |  |  | 0.998 | 90.1 | 0.40 |
|  |  |  | MA | 1× | 1× | 72.4 | 0.92 | 0.975 | 2 | 0.25 | 0.24 | 0.26 | 1.12 | 1.02 | 1.21 | 21.18 | 20.67 | 21.69 |  |  |  |  |  |  | 0.997 | 90.1 | 0.55 |
|  | 1 | 100 | CP | 1× | 2× | 59.3 | 0.56 | 0.975 | 2 | 0.31 | 0.30 | 0.32 | 0.93 | 0.89 | 0.97 | 8.80 | 8.63 | 8.96 |  |  |  |  |  |  | 0.999 | 69.8 | 6.02 |
|  |  |  | MA | 1× | 2× | 53.6 | 0.54 | 0.969 | 2 | 0.47 | 0.46 | 0.48 | 1.01 | 0.97 | 1.05 | 9.83 | 9.51 | 10.17 |  |  |  |  |  |  | 0.998 | 66.5 | 3.19 |
|  | 1 | 100 | CP | 1× | 4× | 41.2 | 0.48 | 0.914 | 2 | 0.59 | 0.58 | 0.59 | 0.44 | 0.43 | 0.45 | 3.51 | 3.40 | 3.62 |  |  |  |  |  |  | 0.999 | 36.8 | 1.60 |
|  |  |  | MA | 1× | 4× | 37.1 | 0.46 | 0.898 | 3 | 0.35 | 0.29 | 0.40 | 0.22 | 0.20 | 0.24 | 5.32 | 4.27 | 6.34 | 0.48 | 0.45 | 0.52 | 0.86 | 0.73 | 0.99 | 0.997 | 30.4 | 3.13 |
|  | 2 | 20.5 | CP | 1× | 1× | 22.0 | 0.21 | 0.981 | 2 | 0.46 | 0.45 | 0.47 | 0.85 | 0.81 | 0.89 | 13.38 | 12.86 | 13.90 |  |  |  |  |  |  | 0.996 | 58.4 | 0.53 |
|  |  |  | MA | 1× | 1× | 21.3 | 0.12 | 0.993 | 2 | 0.42 | 0.40 | 0.44 | 0.83 | 0.77 | 0.89 | 12.27 | 11.66 | 12.89 |  |  |  |  |  |  | 0.993 | 59.2 | 8.29 |
|  | 1 | 100 | CP | 2× | 1× | 60.3 | 0.68 | 0.967 | 2 | 0.35 | 0.34 | 0.36 | 0.73 | 0.69 | 0.76 | 9.34 | 9.14 | 9.55 |  |  |  |  |  |  | 0.998 | 69.1 | 2.27 |
|  |  |  | MA | 2× | 1× | 55.5 | 0.72 | 0.944 | 2 | 0.43 | 0.42 | 0.44 | 0.89 | 0.86 | 0.92 | 9.64 | 9.37 | 9.91 |  |  |  |  |  |  | 0.998 | 69.4 | 2.57 |
|  | 3 | 10.0 | CP | 1/3× | 1/3× | 10.4 | 0.49 | 0.958 | 2^g^ | 0.22 | 0.18 | 0.25 | 2.02 | 0.76 | 3.27 | 118.45 | 105.69 | 131.23 |  |  |  |  |  |  | 0.988 | 100.1 | 7.20 |
|  |  |  | MA | 1/3× | 1/3× | 14.5 | 0.59 | 0.962 | 2^h^ | 0.14 | 0.09 | 0.19 | 0.65 | -0.22 | 1.52 | 82.56 | 73.18 | 91.93 |  |  |  |  |  |  | 0.984 | 101.6 | 7.28 |
| Slip only  Marshall et al. [3,4] | 1 | 100 | CP | 1× | 1/2× | 62.7 | 1.15 | 0.976 | 2^i^ | 0.14 | 0.12 | 0.16 | 2.60 | 1.92 | 3.28 | 37.78 | 36.53 | 39.03 |  |  |  |  |  |  | 0.997 | 99.5 | 0.93 |
|  |  |  | MA | 1× | 1/2× | 61.8 | 1.42 | 0.961 | 2^j^ | 0.24 | 0.14 | 0.34 | 9.62 | 6.50 | 12.73 | 36.63 | 33.03 | 40.23 |  |  |  |  |  |  | 0.997 | 97.6 | 1.91 |
|  | 1 | 100 | CP | 1× | 2/3× | 69.1 | 1.99 | 0.941 | 2^k^ | 0.13 | 0.12 | 0.14 | 1.47 | 1.01 | 1.83 | 29.48 | 28.69 | 30.27 |  |  |  |  |  |  | 0.997 | 96.9 | 2.19 |
|  |  |  | MA | 1× | 2/3× | 55.1 | 1.12 | 0.959 | 2^l^ | 0.15 | 0.13 | 0.17 | 1.18 | 0.88 | 1.48 | 27.51 | 26.61 | 28.41 |  |  |  |  |  |  | 0.997 | 93.4 | 0.89 |
|  | 1 | 100 | CP | 1× | 1× | 56.0 | 1.25 | 0.924 | 2^m^ | 0.18 | 0.17 | 0.19 | 1.03 | 0.92 | 1.14 | 20.46 | 20.09 | 20.84 |  |  |  |  |  |  | 0.998 | 90.3 | 2.46 |
|  |  |  | MA | 1× | 1× | 56.0 | 0.60 | 0.985 | 2^n^ | 0.16 | 0.15 | 0.17 | 1.08 | 0.90 | 1.25 | 23.19 | 22.67 | 23.72 |  |  |  |  |  |  | 0.997 | 90.9 | 0.75 |
|  | 1 | 100 | CP | 1× | 2× | 59.3 | 0.46 | 0.981 | 2 | 0.35 | 0.35 | 0.36 | 0.79 | 0.76 | 0.82 | 9.48 | 9.31 | 9.65 |  |  |  |  |  |  | 0.999 | 71.5 | 4.14 |
|  |  |  | MA | 1× | 2× | 53.6 | 0.54 | 0.969 | 2 | 0.47 | 0.46 | 0.48 | 1.01 | 0.97 | 1.05 | 9.83 | 9.51 | 10.15 |  |  |  |  |  |  | 0.998 | 66.5 | 3.19 |
|  | 1 | 100 | CP | 1× | 4× | 38.7 | 0.18 | 0.985 | 3 | 0.48 | 0.43 | 0.53 | 0.16 | 0.15 | 0.17 | 5.34 | 3.10 | 7.59 | 0.41 | 0.38 | 0.45 | 0.73 | 0.58 | 0.87 | 0.996 | 21.6 | 6.40 |
|  |  |  | MA | 1× | 4× | 29.3 | 0.20 | 0.970 | 3 | 0.60 | 0.55 | 0.65 | 0.13 | 0.12 | 0.14 | 7.95 | 2.01 | 13.89 | 0.33 | 0.29 | 0.37 | 0.65 | 0.47 | 0.82 | 0.994 | 24.3 | 16.44 |
| Catch-slip  Evans et al. [5] | 1 | 8.55E+05 | CP | 1× | 1× | N/A* |  |  | N/A* |  |  |  |  |  |  |  |  |  |  |  |  |  |  |  |  | 104.7 | 0.25 |
|  |  |  | MA | 1× | 1× | N/A* |  |  | N/A* |  |  |  |  |  |  |  |  |  |  |  |  |  |  |  |  | 105.1 | 0.65 |
|  | 2 | 1.12E+03 | CP | 1× | 1× | N/A* |  |  | N/A* |  |  |  |  |  |  |  |  |  |  |  |  |  |  |  |  | 102.1 | 1.24 |
|  |  |  | MA | 1× | 1× | N/A* |  |  | N/A* |  |  |  |  |  |  |  |  |  |  |  |  |  |  |  |  | 101.7 | 0.34 |
|  | 1 | 8.55E+05 | CP | 2× | 1× | N/A* |  |  | N/A* |  |  |  |  |  |  |  |  |  |  |  |  |  |  |  |  | 101.7 | 0.64 |
|  |  |  | MA | 2× | 1× | N/A* |  |  | N/A* |  |  |  |  |  |  |  |  |  |  |  |  |  |  |  |  | 102.3 | 2.22 |
|  | 4 | 39.9 | CP | 1× | 1× | 35.8 | 0.38 | 0.980 | 2 | 0.25 | 0.24 | 0.25 | 0.80 | 0.75 | 0.85 | 14.45 | 14.22 | 14.69 |  |  |  |  |  |  | 0.999 | 80.9 | 6.90 |
|  |  |  | MA | 1× | 1× | 39.6 | 0.41 | 0.981 | 2 | 0.29 | 0.28 | 0.29 | 1.16 | 1.10 | 1.22 | 15.98 | 15.72 | 16.25 |  |  |  |  |  |  | 0.999 | 79.1 | 6.22 |

*Not enough pause events detected to perform a statistical analysis.

^a^ Results passed the likelihood ratio test for the 2-component Poisson at the 85th percentile, but qualitatively the results were much improved with the 3-component Poisson estimate. The sample size was about 250 skip events.

^b^ Results passed the likelihood ratio test for the 1-component Poisson at the 91st percentile, but qualitatively the results were much improved with the 2-component Poisson estimate. The sample size was 80 skip events.

^c^ Results passed the likelihood ratio test for the 1-component Poisson at the 74th percentile, but qualitatively the results were much improved with the 2-component Poisson estimate. The sample size was 76 skip events.

^d^ Results passed the likelihood ratio test for the 1-component Poisson at the 67th percentile, but qualitatively the results were much improved with the 2-component Poisson estimate. The sample size was 97 skip events.

^e^ Results passed the likelihood ratio test against the 1-component Poisson at the 62nd percentile, but qualitatively the results were slightly improved with the 2-component Poisson estimate. The sample size was 88 skip events.

^f^ Results passed the likelihood ratio test for the 1-component Poisson at the 79th percentile, but qualitatively the results were improved with the 2-component Poisson estimate, especially for short skips. The sample size was 129 skip events.

^g^ Results passed the likelihood ratio test for the 1-component Poisson at the 75th percentile, but qualitatively the results were significantly improved with the 2-component Poisson estimate. The sample size was only 24 skip events due to the low site density.

^h^ Results passed the likelihood ratio test for the 1-component Poisson at the 88th percentile, but qualitatively the results were significantly improved with the 2-component Poisson estimate. The sample size was only 29 skip events due to the low site density.

^i^ Results passed the likelihood ratio test for the 1-component Poisson at the 68th percentile, but qualitatively the fit was significantly improved with the 2-component Poisson estimate. The sample size was 81 skip events.

^j^ Results passed the likelihood ratio test for the 1-component Poisson at the 52nd percentile, but qualitatively the fit was significantly improved with the 2-component Poisson estimate. The sample size was 89 skip events.

^k^ Results passed the likelihood ratio test for the 1-component Poisson at the 80th percentile, but qualitatively the fit was improved with the 2-component Poisson estimate. The sample size was 99 skip events.

^l^ Results passed the likelihood ratio test for the 1-component Poisson at the 60th percentile, but qualitatively the fit was better with the 2-component Poisson estimate. The sample size was 118 skip events.

^m^ Results passed the likelihood ratio test for the 1-component Poisson at the 78th percentile, but qualitatively the fit was significantly improved with the 2-component Poisson estimate. The sample size was 147 skip events.

^n^ Results passed the likelihood ratio test for the 1-component Poisson at the 76th percentile, but qualitatively the fit was significantly improved with the 2-component Poisson estimate. The sample size was 136 skip events.

**References**

1. Park EY, Smith MJ, Stropp ES, Snapp KR, DiVietro JA, et al. (2002) Comparison of PSGL-1 microbead and neutrophil rolling: microvillus elongation stabilizes P-selectin bond clusters. Biophys J 82: 1835-1847.

2. Edmondson KE, Denney WS, Diamond SL (2005) Neutrophil-bead collision assay: pharmacologically induced changes in membrane mechanics regulate the PSGL-1/P-selectin adhesion lifetime. Biophys J 89: 3603-3614.

3. Marshall BT, Long M, Piper JW, Yago T, McEver RP, et al. (2003) Direct observation of catch bonds involving cell-adhesion molecules. Nature 423: 190-193.

4. Beste MT, Hammer DA (2008) Selectin catch-slip kinetics encode shear threshold adhesive behavior of rolling leukocytes. Proc Natl Acad Sci USA 105: 20716-20721.

5. Evans E, Leung A, Heinrich V, Zhu C (2004) Mechanical switching and coupling between two dissociation pathways in a P-selectin adhesion bond. Proc Natl Acad Sci USA 101: 11281-11286.
