# Supplementary material for: Nano-motion Dynamics are Determined by Surface-Tethered Selectin Mechanokinetics and Bond Formation
Source: PLoS Comput Biol. 2009 Dec 18;5(12):e1000612. doi: 10.1371/journal.pcbi.1000612 (PMC2787012; doi:10.1371/journal.pcbi.1000612)
Supplement: Figure S1 — Assumed models of force extension. The employed models of molecular elongation with force are compared to experimental data and alternatives. (0.06 MB DOC) [file pcbi.1000612.s004.doc]

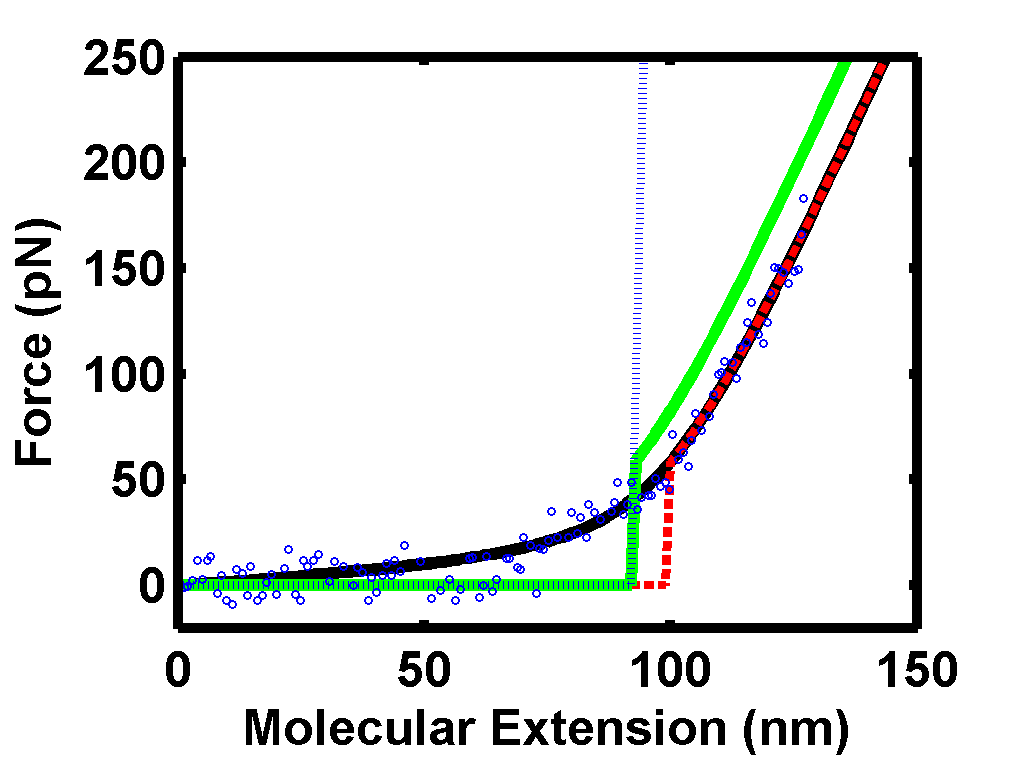


**Figure S1. Assumed models of force extension.**

Blue dots illustrated data from the study by Fritz et al. for P-selectin/PSGL-1 [1]. **The heavy black line** illustrates the freely-jointed chain model with parameters measured by Fritz et al. [1]. The dashed red line illustrates the same model modified with a step superimposed at the contour measured by Fritz et al. [1]. **The heavy green line** illustrates the modified freely-jointed chain model employed in the simulation with a 92 nm contour length. The step served to simplify the model so molecular extension probabilities imposed by energetic constraints would not need to be considered in bond formation kinetics. The model still matched the data reported in the study by Fritz et al. [1] well. The blue dotted line illustrates the rope model. As with the modified freely-jointed chain model, bonds are unstressed until they reach their contour length. Rope bonds stretched beyond their contact length behave as a very stiff spring.

1. Fritz J, Katopodis AG, Kolbinger F, Anselmetti D (1998) Force-mediated kinetics of single P-selectin/ligand complexes observed by atomic force microscopy. Proc Natl Acad Sci USA 95: 12283-12288.
