# Supplementary material for: Nano-motion Dynamics are Determined by Surface-Tethered Selectin Mechanokinetics and Bond Formation
Source: PLoS Comput Biol. 2009 Dec 18;5(12):e1000612. doi: 10.1371/journal.pcbi.1000612 (PMC2787012; doi:10.1371/journal.pcbi.1000612)
Supplement: Figure S2 — P-selectin/PSGL-1 bond lifetimes with alternative force dissociation models, parameters, and valencies. The bond dissociation rate as a function of force is shown for several published measurements of P-selectin/PSGL-1 bonds. (0.08 MB DOC) [file pcbi.1000612.s005.doc]

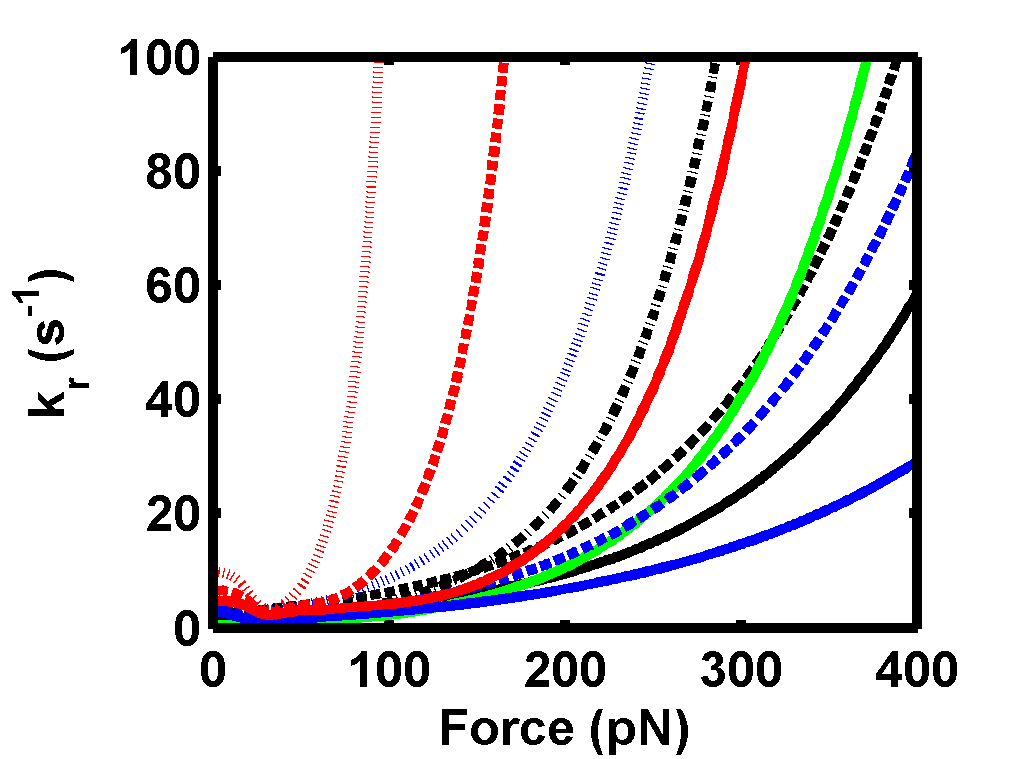


**Figure S2. P-selectin/PSGL-1 bond lifetimes with alternative force dissociation models, parameters, and valencies.**

The bond dissociation rate as a function of force is shown for several published measurements of P-selectin/PSGL-1 bonds. The black lines illustrate Bell model dissociation parameters from various flow cell studies. Black dot-dashed line: cells from Chen and Springer [1]; black dashed line: cells from Smith et al. [2]; black solid line: microbeads from Park et al. [3]. The red lines illustrate estimated catch-slip parameters from the discussion of the biomembrane force probe results from Evans et al. [4], with different valency assumptions from reliability theory. Red dotted line: single-bond cluster; red dashed line: double-bond clusters; red solid line: quadruple-bond clusters. The blue lines illustrate results from the flow cell studies by Marshall et al. [5], but with the five parameter model regression values reported by Beste and Hammer [6]. The study design suggests data was primarily for dimers. Blue dotted line: single-dimer cluster; blue dashed line: double-dimer cluster; blue solid line: triple-dimer cluster. The solid green line illustrates bond dissociation rates for a Bell model dissociation using dimeric groupings of the parameters from the optimization of Edmondson et al. [7]. Note that the k_r_ shown is the statistical point estimate calculated with method of Edmondson et al. [7], which can account for multivalency assumptions of a unit parameter set (e.g. blue dashed line, blue solid line, red dashed line, red solid line, green line).

**References**

1. Chen S, Springer TA (2001) Selectin receptor-ligand bonds: Formation limited by shear rate and dissociation governed by the Bell model. Proc Natl Acad Sci U S A 98: 950-955.

2. Smith MJ, Berg EL, Lawrence MB (1999) A direct comparison of selectin-mediated transient, adhesive events using high temporal resolution. Biophys J 77: 3371-3383.

3. Park EY, Smith MJ, Stropp ES, Snapp KR, DiVietro JA, et al. (2002) Comparison of PSGL-1 microbead and neutrophil rolling: microvillus elongation stabilizes P-selectin bond clusters. Biophys J 82: 1835-1847.

4. Evans E, Leung A, Heinrich V, Zhu C (2004) Mechanical switching and coupling between two dissociation pathways in a P-selectin adhesion bond. Proc Natl Acad Sci USA 101: 11281-11286.

5. Marshall BT, Long M, Piper JW, Yago T, McEver RP, et al. (2003) Direct observation of catch bonds involving cell-adhesion molecules. Nature 423: 190-193.

6. Beste MT, Hammer DA (2008) Selectin catch-slip kinetics encode shear threshold adhesive behavior of rolling leukocytes. Proc Natl Acad Sci USA 105: 20716-20721.

7. Edmondson KE, Denney WS, Diamond SL (2005) Neutrophil-bead collision assay: pharmacologically induced changes in membrane mechanics regulate the PSGL-1/P-selectin adhesion lifetime. Biophys J 89: 3603-3614.
