# Supplementary material for: Nano-motion Dynamics are Determined by Surface-Tethered Selectin Mechanokinetics and Bond Formation
Source: PLoS Comput Biol. 2009 Dec 18;5(12):e1000612. doi: 10.1371/journal.pcbi.1000612 (PMC2787012; doi:10.1371/journal.pcbi.1000612)
Supplement: Figure S3 — Velocities using the Bell model dissociation parameters of Park et al. Instantaneous velocity results from simulations utilizing the dissociation kinetics reported by Park et al. are presented as a function of time. (0.84 MB DOC) [file pcbi.1000612.s006.doc]

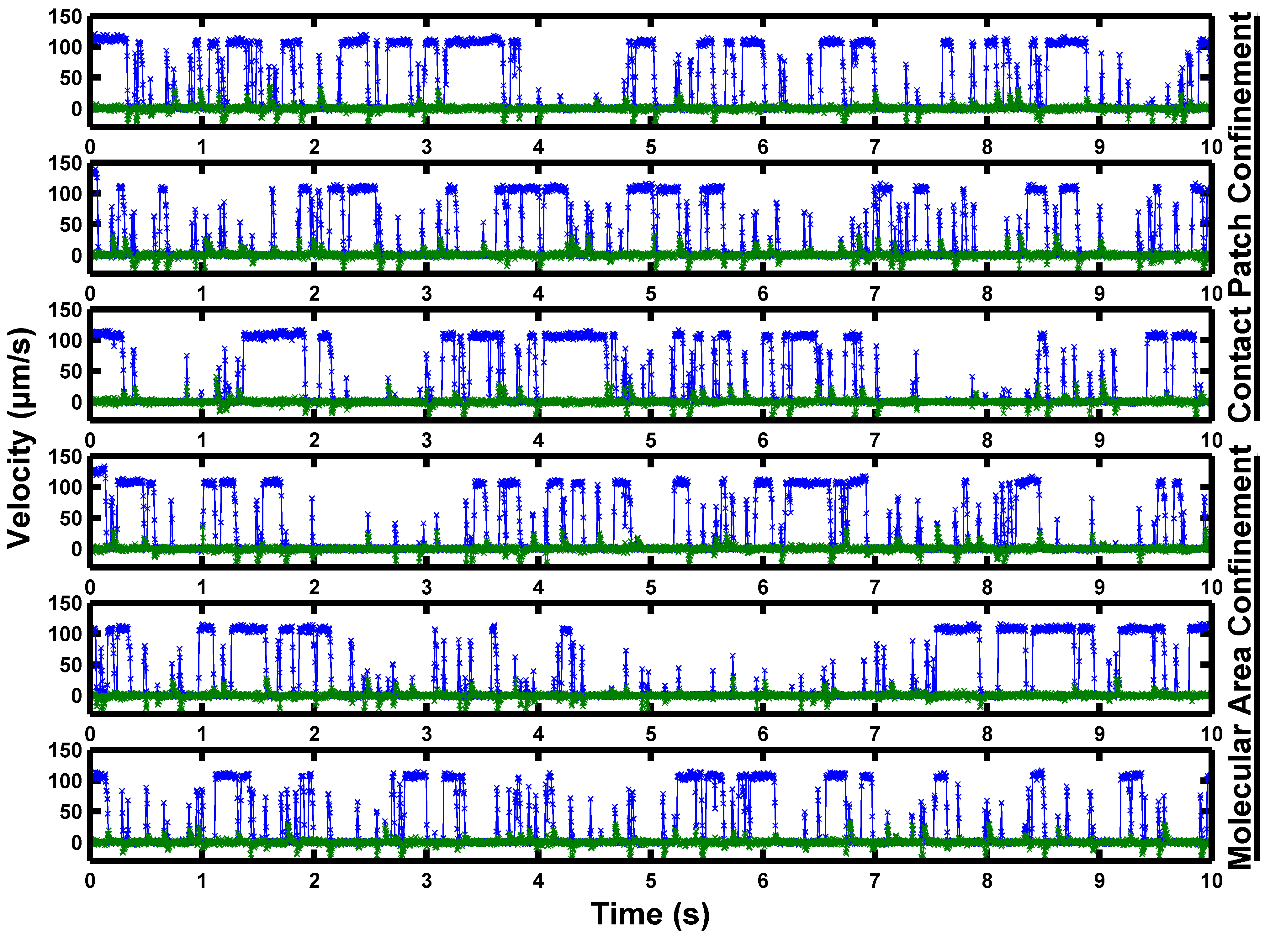


**Figure S3. Velocities using the Bell model dissociation parameters of Park et al. [1].**

Simulations were run using the site densities, sphere diameter, and wall shear rate from the study by Park et al. [1] and with the Bell model dissociation parameters measured in the same study. The conditions were: S=50 s^-1^, R=4.9 µm, n_L_º=90 sites/µm^2^, and n_R_º=95 sites/µm^2^. The blue lines indicate the instantaneous sampled flow-direction velocity, V_S,X_, and the green lines indicate the perpendicular velocity, V_S,Y_. Velocities were sampled at 250 fps.

**References**

1. Park EY, Smith MJ, Stropp ES, Snapp KR, DiVietro JA, et al. (2002) Comparison of PSGL-1 microbead and neutrophil rolling: microvillus elongation stabilizes P-selectin bond clusters. Biophys J 82: 1835-1847.
