# Supplementary material for: Nano-motion Dynamics are Determined by Surface-Tethered Selectin Mechanokinetics and Bond Formation
Source: PLoS Comput Biol. 2009 Dec 18;5(12):e1000612. doi: 10.1371/journal.pcbi.1000612 (PMC2787012; doi:10.1371/journal.pcbi.1000612)
Supplement: Figure S4 — Velocities using the five-parameter catch-slip model dissociation parameters derived from the experiments of Marshall et al. Instantaneous velocity results from simulations utilizing the dissociation kinetics from the study by Marshall et al. are presented as a function of time. (0.94 MB DOC) [file pcbi.1000612.s007.doc]

**
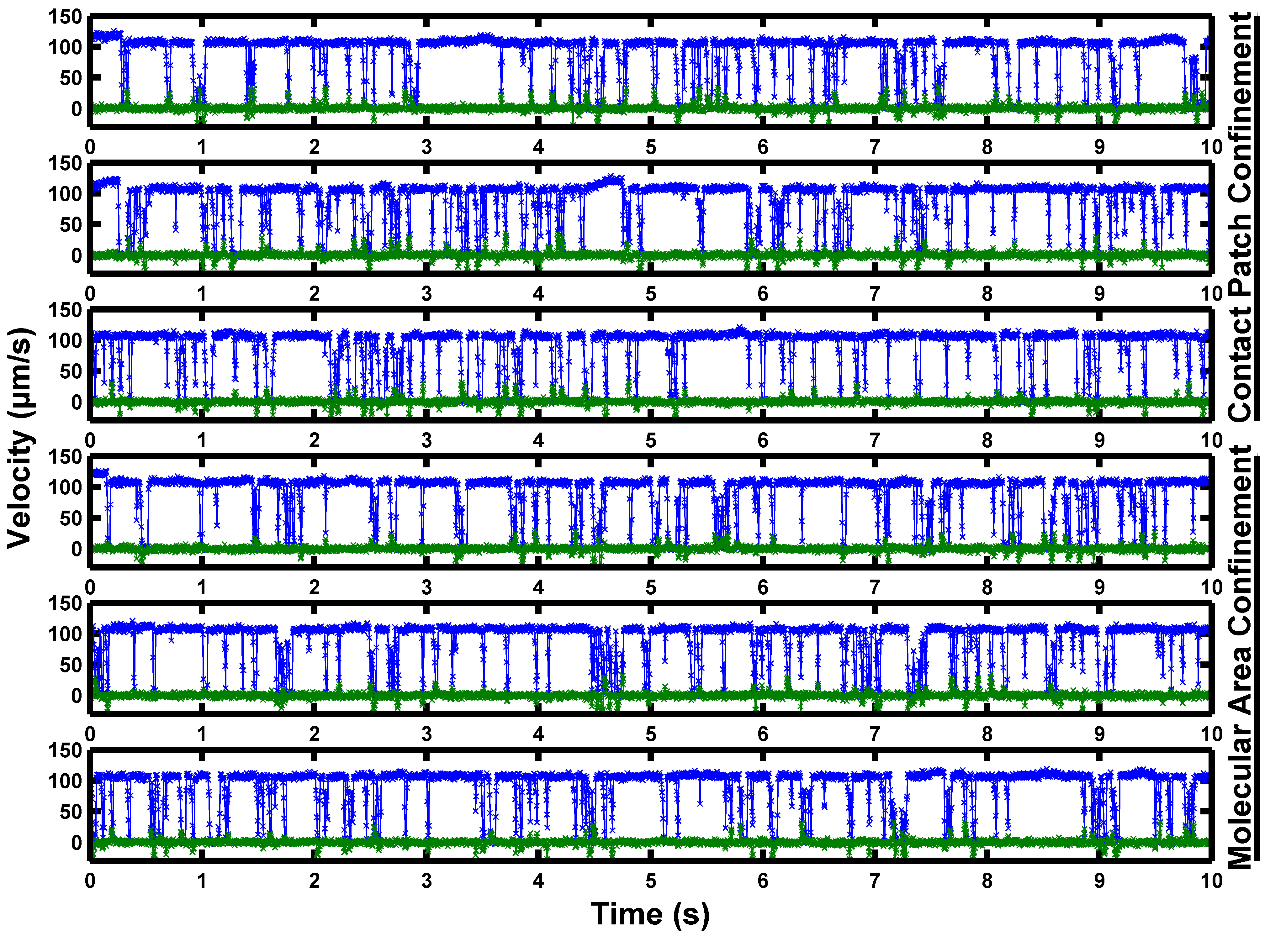
**

**Figure S4. Velocities using the five-parameter catch-slip model dissociation parameters derived from the experiments of Marshall et al. [1].**

Simulations were run using the site densities, sphere diameter, and wall shear rate from the study by Park et al. [2] and with the dimeric catch-slip dissociation parameters from Marshall et al. [1], as reported in the regression by Beste and Hammer [3]. The conditions were: S=50 s^-1^, R=4.9 µm, n_L_º=90 sites/µm^2^, and n_R_º=95 sites/µm^2^. The blue lines indicate the instantaneous sampled flow-direction velocity, V_S,X_, and the green lines indicate the perpendicular velocity, V_S,Y_. Velocities were sampled at 250 fps.

**References**

1. Marshall BT, Long M, Piper JW, Yago T, McEver RP, et al. (2003) Direct observation of catch bonds involving cell-adhesion molecules. Nature 423: 190-193.

2. Park EY, Smith MJ, Stropp ES, Snapp KR, DiVietro JA, et al. (2002) Comparison of PSGL-1 microbead and neutrophil rolling: microvillus elongation stabilizes P-selectin bond clusters. Biophys J 82: 1835-1847.

3. Beste MT, Hammer DA (2008) Selectin catch-slip kinetics encode shear threshold adhesive behavior of rolling leukocytes. Proc Natl Acad Sci USA 105: 20716-20721.
