# Supplementary material for: Nano-motion Dynamics are Determined by Surface-Tethered Selectin Mechanokinetics and Bond Formation
Source: PLoS Comput Biol. 2009 Dec 18;5(12):e1000612. doi: 10.1371/journal.pcbi.1000612 (PMC2787012; doi:10.1371/journal.pcbi.1000612)
Supplement: Figure S5 — Velocities using the five-parameter catch-slip model dissociation parameters of Evans et al. Instantaneous velocity results from simulations utilizing the dissociation kinetics from the study by Evans et al. are presented as a function of time. (0.65 MB DOC) [file pcbi.1000612.s008.doc]

**
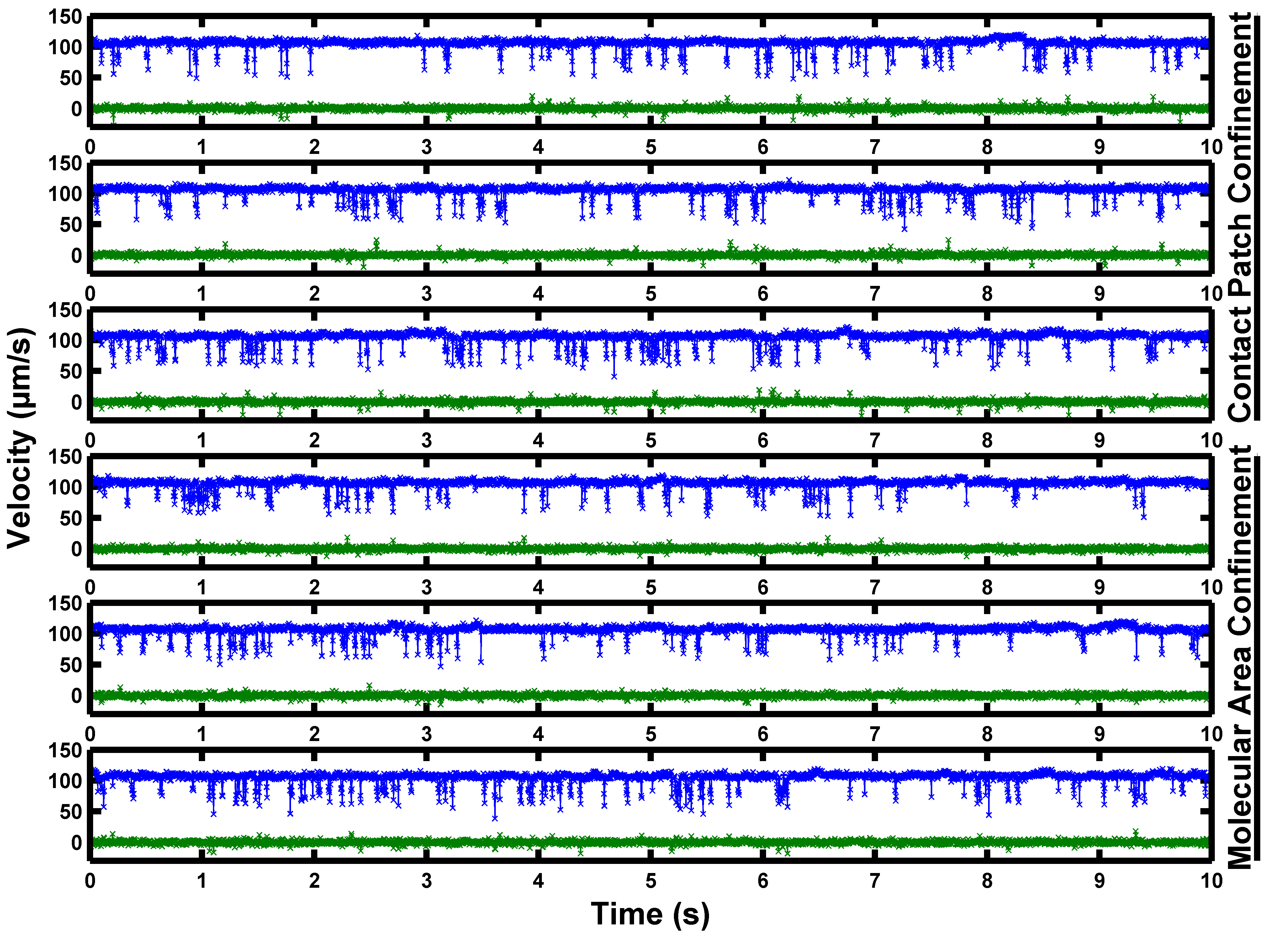
**

**Figure S5. Velocities using the five-parameter catch-slip model dissociation parameters of Evans et al. [1].**

Simulations were run using the site densities, sphere diameter, and wall shear rate from the study by Park et al. [2] and with the catch-slip dissociation parameters from Evans et al. [1]. The conditions were: S=50 s^-1^, R=4.9 µm, n_L_º=90 sites/µm^2^, and n_R_º=95 sites/µm^2^. The blue lines indicate the instantaneous sampled flow-direction velocity, V_S,X_, and the green lines indicate the perpendicular velocity, V_S,Y_. Velocities were sampled at 250 fps.

**References**

1. Evans E, Leung A, Heinrich V, Zhu C (2004) Mechanical switching and coupling between two dissociation pathways in a P-selectin adhesion bond. Proc Natl Acad Sci USA 101: 11281-11286.

2. Park EY, Smith MJ, Stropp ES, Snapp KR, DiVietro JA, et al. (2002) Comparison of PSGL-1 microbead and neutrophil rolling: microvillus elongation stabilizes P-selectin bond clusters. Biophys J 82: 1835-1847.
