# Supplementary material for: Nano-motion Dynamics are Determined by Surface-Tethered Selectin Mechanokinetics and Bond Formation
Source: PLoS Comput Biol. 2009 Dec 18;5(12):e1000612. doi: 10.1371/journal.pcbi.1000612 (PMC2787012; doi:10.1371/journal.pcbi.1000612)
Supplement: Figure S6 — A model of encounter complex formation and bond formation. A model accounting for the role of both the molecular tether and binding pocket chemistry in bond formation is presented. The model can be employed with the assumption that the receptor and ligand are each confined by an immobile tether anchor point to a separate surface. (0.16 MB DOC) [file pcbi.1000612.s009.doc]

**A No Complex B Tether-Point Encounter Complex**

**D Bond C Binding-Pocket Encounter Complex**


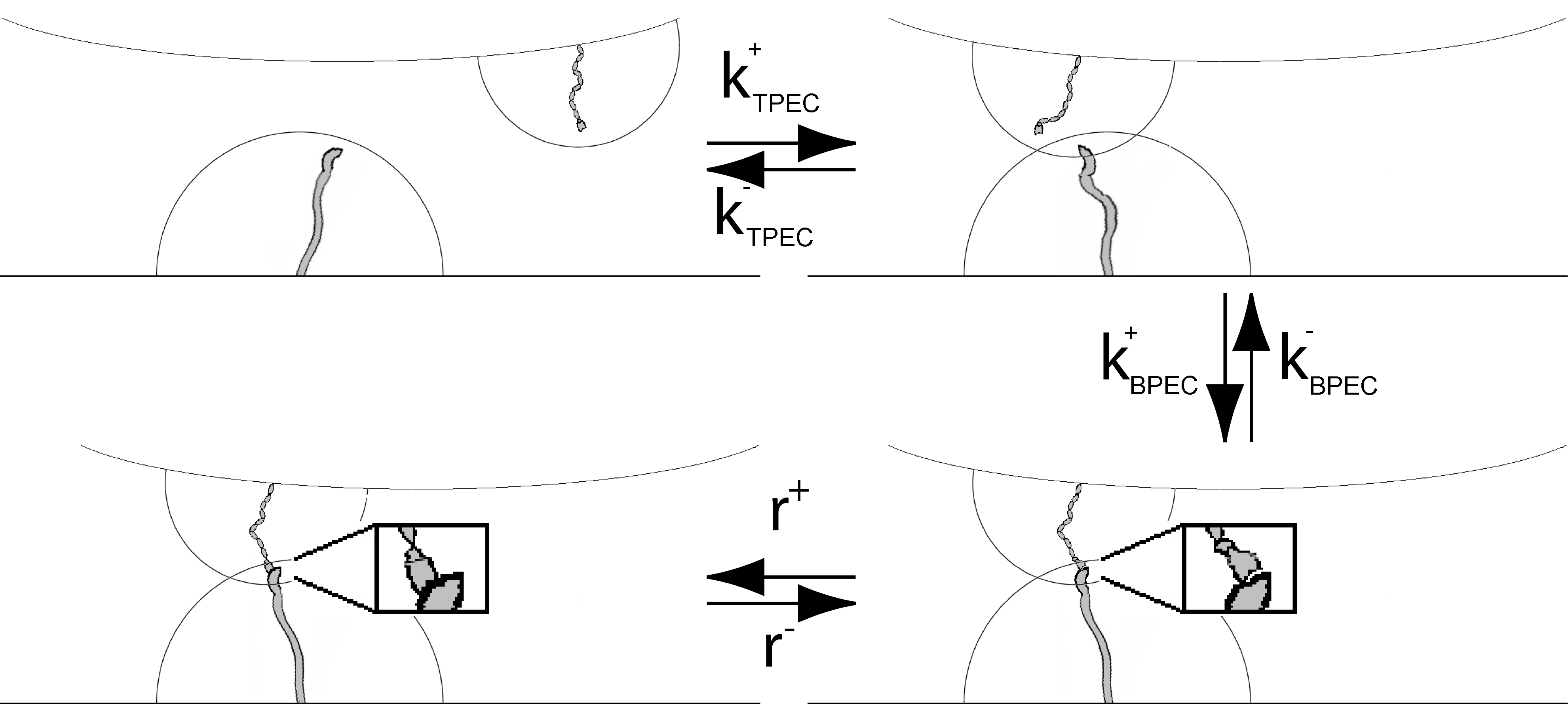


**Figure S6. A model of encounter complex formation and bond formation.**

(A) A receptor on the sphere is not within binding distance of any ligands on the functionalized surface. (B) The rotational and translational motion of the sphere brings the receptor and ligand closer together. The points where they are tethered to their respective surfaces are within a non-mechanically strained, maximum extended molecular length of each other. This is a tether-point encounter complex. If enough ligands were immobilized on the surface, the receptor on the sphere might be able to engage in multiple tether-point encounter complexes simultaneously. Motion of the sphere can also carry the receptor away from reactive ligands. (C) The motion of the receptor’s reactive head group on its molecular stalk enables it to approach the reactive site on the ligand. This state is the binding-pocket encounter complex. Such encounters might result in a rapid transition into a non-covalent biomolecular bond that can resist a mechanical pulling force. Alternatively, such encounters might happen frequently with a suboptimal orientation that results in the binding-pocket encounter complex reverting back to a tether-point encounter complex. A receptor can only engage in one binding-pocket encounter complex at a time. (D) A stable bond that can resist a mechanical pulling force forms.
